# Dietary α-Linolenic Acid-Rich Flaxseed Oil Ameliorates High-Fat Diet-Induced Atherosclerosis *via* Gut Microbiota-Inflammation-Artery Axis in *ApoE*^−/−^ Mice

**DOI:** 10.3389/fcvm.2022.830781

**Published:** 2022-02-28

**Authors:** Yiwei Li, Zhi Yu, Yuanyuan Liu, Ting Wang, Yajuan Liu, Zhixia Bai, Yi Ren, Huiyan Ma, Ting Bao, Haixia Lu, Rui Wang, Libo Yang, Ning Yan, Ru Yan, Shaobin Jia, Xiaoxia Zhang, Hao Wang

**Affiliations:** ^1^School of Basic Medical Sciences, Ningxia Medical University, Yinchuan, China; ^2^Department of Anesthesiology, People's Hospital of Ningxia Hui Autonomous Region, Yinchuan, China; ^3^Clinical Medical College, Ningxia Medical University, Yinchuan, China; ^4^Department of Cardiovascular Diseases, Heart Centre, General Hospital of Ningxia Medical University, Yinchuan, China; ^5^Ningxia Key Laboratory of Vascular Injury and Repair Research, Ningxia Medical University, Yinchuan, China; ^6^College of Traditional Chinese Medicine, Ningxia Medical University, Yinchuan, China

**Keywords:** ALA-rich flaxseed oil, atherosclerosis, inflammation, gut microbiota, intestinal metabolites

## Abstract

Atherosclerosis (AS) is closely associated with abnormally chronic low-grade inflammation and gut dysbiosis. Flaxseed oil (FO) rich in omega-3 polyunsaturated fatty acids (PUFAs), which are mainly composed of alpha-linolenic acid (ALA, 18:3 omega-3), has been demonstrated to exhibit pleiotropic benefits in chronic metabolic diseases. However, the impact of dietary ALA-rich FO on AS and its associated underlying mechanisms remain poorly understood. Thus, the present study was designed as two phases to investigate the effects in atherosclerotic *Apolipoprotein E* (*ApoE*)^−/−^ mice. In the initial portion, the *ApoE*^−/−^ mice were randomly allocated to three groups: control group (CON), model group (MOD), and FO-fed model group (MOD/FO) and were treated for 12 weeks. The second phase used antibiotic (AB)-treated *ApoE*^−/−^ mice were divided into two groups: AB-treated model group (AB/MOD) and FO-fed AB-treated model group (AB/FO). In the results, the dietary ALA-rich FO administration ameliorated atherosclerotic lesion, as well as the parameters of AS (body weights (BWs) and the total bile acids (TBA). Chronic systemic/vascular inflammatory cytokines and *in situ* macrophages (Mψs) were reduced with FO intervention. In addition, the FO improved the gut integrity and permeability by decreasing the plasma lipopolysaccharide (LPS). Moreover, gut dysbiosis and metabolites [short-chain fatty acids (SCFAs) and bile acids (BAs)] in AS were modulated after FO treatment. Intriguingly, during an AB-treated condition, a significantly weakened amelioration of FO-treated on AS proposed that the intestinal microbiota contributed to the FO effects. A correlation analysis showed close relationships among gut bacteria, metabolites, and inflammation. Collectively, these results suggested that the dietary ALA-rich FO ameliorated the AS in *ApoE*^−/−^ mice *via* the gut microbiota-inflammation-artery axis.

## Introduction

Atherosclerosis (AS), characterized by excessive cholesterol deposition within the artery wall, is the leading cause of cardiovascular diseases (CVD) including coronary heart disease, cerebral infarction myocardial infarction (MI), stroke, and peripheral vascular disease (1), accounting for a major cause of mortality and morbidity in the world (2, 3). Moreover, AS represents a chronic inflammatory disorder of the vascular wall involving many circulating immune cells, such as monocytes, lymphocytes, and platelets (4). Among them, the monocyte infiltration and the subsequent formation of macrophages (Mψs)-derived foam cells for the release of pro-inflammatory cytokines are crucial in the progression of AS (1, 5, 6). Monocytes are rich in cell-activating, oxidized, and low-density lipoprotein (oxLDL) in accumulating the development of lesions and forming the early plaques (known as fatty streaks) on the intima (7). The AS changes from fatty streaks to plaque rupture as well as thrombosis. Due to limited dietary management of AS, some novel strategies are urgently needed.

Chronic inflammation has been involved in the occurrence and the development of AS (8, 9). During the pathological injury of AS, a variety of pro-inflammatory cytokines, including the tumor necrosis factor (TNF)-α, the interleukin (IL)-1β, and IL-6 were increased (10). It was verified that a *Toll-like receptor* (*Tlr4*)^−/−^ and/or *myeloid differentiation factor* (*Myd*)*88*^−/−^ have declined the production of inflammatory cytokines in *Apolipoprotein E* (*ApoE*)^−/−^ mice, thereby reducing the formation of aortic plaque (11). Furthermore, inflammation was attenuated by inhibiting the Jun N-terminal kinase (JNK) and the nuclear factor kappa-B (NF-κB) pathways in atherosclerotic *LDL*^−/−^ mice (12). Consistently, clinical studies have shown a significantly increased inflammation in the AS lesion (13).

The monocytes/Mψs represent the key inflammatory cells in AS. Instability and rupture of atherosclerotic plaques occur predominantly in regions where inflammatory monocytes/Mψs adhere to the plaque shoulder areas (14). As a trigger of Mψ-mediated inflammation, lipopolysaccharide (LPS) from Gram-negative bacteria binds to TLR-4 and promotes the pro-inflammatory cytokines generation and release, which ultimately erodes the extracellular matrix and leads to the plaque rupture followed by MI or stroke (15, 16).

Gut microbiota plays a vital role in the progression of AS (17). Emerging studies have suggested that gut dysbiosis provokes the damage of intestinal mucosal barriers and enhances the intestinal permeability, thus, leading to the translocations of pathogenic bacteria and their metabolites (such as LPS) into the plasma *via* the gut-heart axis, for triggering blood vessel chronic inflammation (18). As crucial metabolites of gut microorganisms, short-chain fatty acids (SCFAs), with <6 carbon atoms (C1-C6) and mainly include acetate (C2), propionate (C3), and butyrate (C4), are essential for intestinal homeostasis (19). Importantly, a remarkable reduction of SCFAs led to a dysfunction of the gut mucosal barrier in AS (20). In addition, another microbial metabolite, the bile acids (BAs), especially the secondary BAs (SBAs), act as ligands that activated the BA-activated G protein-coupled receptor 1 (GP-BAR1) to stabilize the intestinal barrier function (21). Besides, the BAs exert various functions on intestinal lipid absorption and metabolic regulation. They were identified as the specific microbial enzymatic participants, which in turn, modulated the composition of the microbiota (22).

The flaxseed oil (FO), rich in plant-derived omega-3 (ω-3) polyunsaturated fatty acids (PUFAs) and mainly α-linolenic acid (ALA, 18:3 ω-3) (23), has been demonstrated by researchers, including our lab to exhibit pleiotropic benefits in chronic metabolic diseases, such as alcoholic liver disease (ALD) (23), polycystic ovarian syndrome (PCOS) (24), and type 2 diabetes mellitus (T2DM) (25) and colitis (26). However, the effects of the dietary ALA-rich FO on AS and the underlying mechanisms remain elusive (27). Thus, this study was aimed to investigate the effectiveness of the dietary ALA-rich FO on the occurrence and the development of AS in *ApoE*^−/−^ mice, with or without gut microbiota, which may potentially contribute to the further understanding of complicated mechanisms among AS, gut microbiota, and inflammation.

## Materials and Methods

### Animals and Diets

All experiments were approved by the Ethics Committee of Ningxia Medical University (No. 2019-137). Fifty male *ApoE*^−/−^ mice (8-week-old) were obtained from Vital River Laboratory Animal Technology Co., Ltd., Beijing, China. The mice were maintained under standard, specific, and pathogen-free conditions in individual cages in a temperature-controlled room (ambient temperature 22 ± 1°C, air humidity 40–70%) with a 12 h light/dark cycle room in Laboratory Animal Center of Ningxia Medical University, Yinchuan, China. A high-fat diet (HFD) with 1.25% cholesterol (60% fat, 20% carbohydrate, and 20% protein, No. TP28520) was purchased from TROPHIC Animal Feed High-tech Co., Ltd., Nantong, China. The FO with 59.58 ± 2.47% ALA was extracted by our laboratory.

### Experimental Design

**Figure 1:**
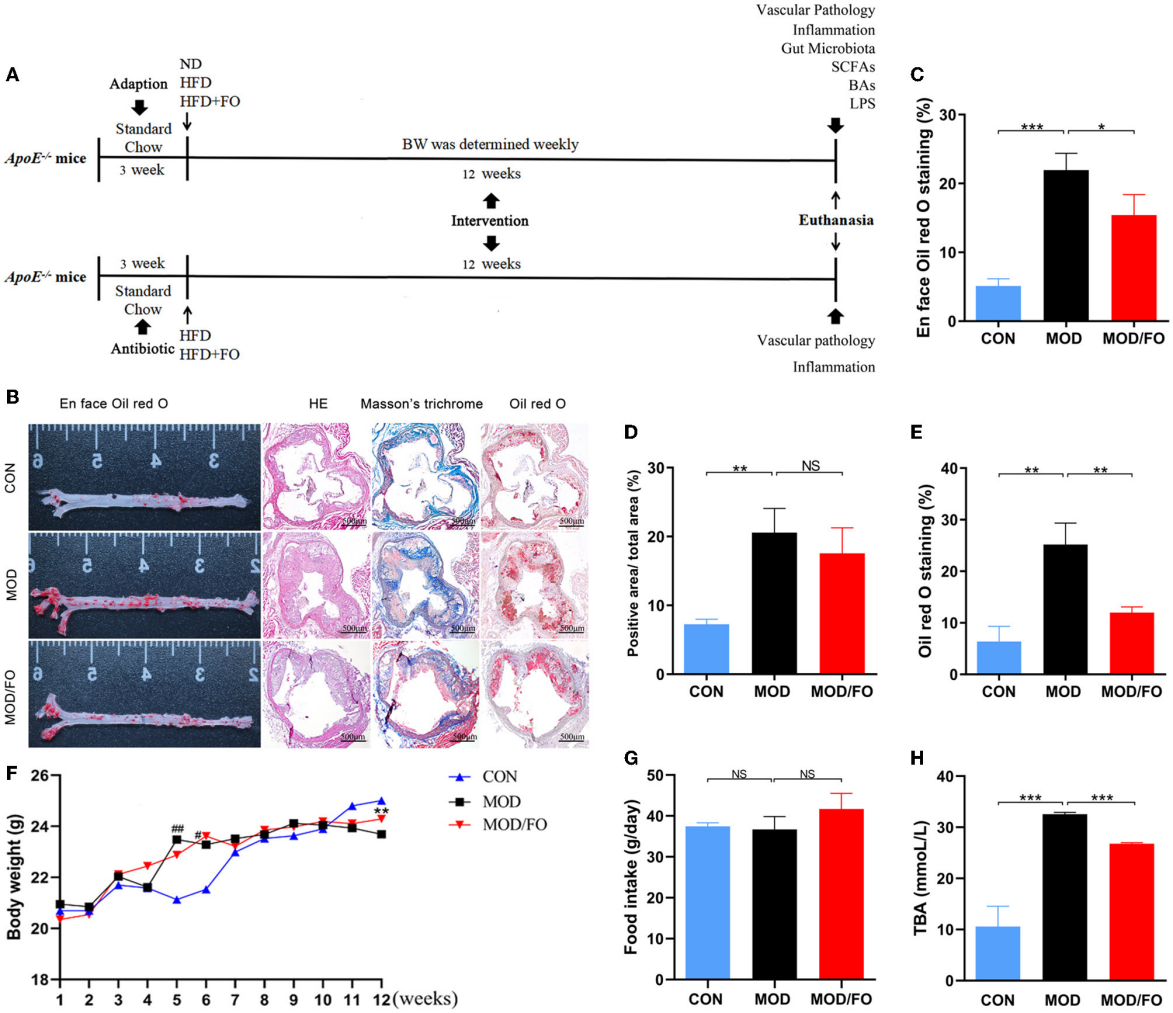
Effects of dietary ALA-rich FO treatment on the pathological lesion and routine parameters in atherosclerotic *ApoE*^−/−^ mice. **(A)** Schematic time diagram of the experimental design. **(B)** Representative stained sections of the valve area of the aorta and aortic root of the heart. Quantitative analysis as lesion area/total area (%) shown in *en* face oil-red O staining (*n* = 5/group) **(C)**, Masson's trichrome staining **(D)**, and oil-red O staining **(E)**. **(F)** BWs: Body weights. **(G)** Food intake. **(H)** TBA: total bile acid. Original magnification, ×40. The bar of 500 μm was presented in the right corner of **(B)**. Data were presented as mean ± SEM. ^#^*P* < 0.05, ^*##*^*P* < 0.01, CON group vs. MOD group. *P < 0.05, **P < 0.01, ***P < 0.001, MOD group vs. MOD/FO group. NS, no significance.

As shown in Figure 1A, the experiment was divided into two phases. In the initial portion, after 3 weeks of acclimatization period, the male *ApoE*^−/−^ mice (*n* = 36, 8 weeks old) were randomly divided into 3 groups (12 animals/group): (a) control group (CON), mice were fed a normal diet; (b) model group (MOD), mice were fed HFD with 1.25% cholesterol (w/w); and (c) MOD-treated with ALA-rich FO group (MOD/FO), MOD mice were fed with 10% FO (w/w) as FO intervention group. The mice in MOD groups were fed with the HFD, with an energy composition of 60% fat, 20% carbohydrate, and 20% protein. Meanwhile, the mice in the MOD/FO group received an equal amount of calories as the MOD group with the cocoa butter-derived calories that were substituted with isocaloric FO (fat). The HFD was prepared by the company in advance and was stored at 4°C. After 12 weeks of FO treatment, we fasted the mice for 12 h, and then euthanized them for further analysis. In the second phase, according to previous reports (28), after 3 weeks of antibiotic (AB) cocktail (0.5 g/L vancomycin, 1 g/L neomycin sulfate, 1 g/L metronidazole, and 1 g/L ampicillin), the AB-treated male *ApoE*^−/−^ mice (*n* = 24) were randomly divided into two groups (12 animals/group): MOD group (AB/MOD) and MOD-treated with FO group (AB/FO). After 12 weeks of HFD feeding, the mice were fasted for 12 h and then euthanized for further studies in consistence with the initial period of this study.

### Histology and Morphometry Evaluations of Atherosclerotic Lesions

The pathological changes in AS were detected with *en* face oil-red O staining, HE staining, and Masson's trichrome staining (*n* = 5/group) (Details in Supplementary Material). Images were captured with Canon EOS 70D camera and were analyzed using Image J 1.8.0 software (National Institutes of Health, United States). The lesion area index was calculated as the percentage of aortic lumen area covered by atherosclerotic lesions. Observers were blinded to the experimental groups.

### Determination of Total Bile Acids (TBA) in Serum

The levels of TBA in plasma were measured by AU400 automatic biochemical analyzer (Olympus, Japan).

### Cytometric Bead Array (CBA)

The aorta tissues (100 mg) were homogenized by a glass grinder. After centrifugation at 300 × *g* for 5 min, the supernatants of homogenates were collected to determine the aortic levels of IL-1β, TNF-α, IL-6, IL-10, IL-17A, and MCP-1. These plasma and aortic inflammatory cytokines in each group were measured by LEGENDplex™ CBA mouse inflammatory cytokine kit (Biolegend, United States, No. 740150). The operation was performed three times according to the manufacturer's instructions. In brief, a series of cytokine standard dilutions were prepared. After that, 25 μl of Assay Buffer, 25 μl of each standard, 25 μl of the plasma/supernatants of aortic samples or standard dilutions of each sample, and 25 μl of mixed beads were added to all the wells in turn. All the tubes were placed on a plate shaker, shaking it at ~500 revolutions per minute (rpm) for 2 h at room temperature. The samples were washed with 200 μl wash buffer and centrifuged at 200 × *g* for 5 min. About 25 μl of detection antibodies were added to each well after discarding the supernatant. All the tubes were placed on a plate shaker and were shook at ~500 rpm for 1 h at room temperature. About 25 μl of Streptavidin-Phycoerythrin (SA-PE) was added to each well directly. The plate was placed on a plate shaker and shook at ~500 rpm for 30 min at room temperature. Next, 150 μl of 1 × Wash Buffer were added to each well to shake them for 1 min. Afterward, the flow cytometer was sited with cytometer setup beads and the samples were measured. The data were analyzed by using LEGENDplex software (Biolegend, United States).

### Flow Cytometry Analysis

The Mψs, the significant aortic inflammatory cells (4), were digested and isolated from aortic tissues. Briefly, 1 g of aortic tissues was minced and suspended in 5 ml of Hanks balanced salt solution (HBSS) containing 0.1% (w/v) collagenase type IV (Sigma, United States) for 20 min at 37°C. Next, the specimen was washed with RPMI1640 containing 2% of fetal bovine serum (FBS) and then filtered through a 200-mesh nylon membrane. After centrifugation at 70 × *g* for 3 min at 4°C, the supernatants were discarded, and the particles were resuspended in 3 ml HBSS. After the erythrocyte lysis, samples were centrifuged for 5 min at 500 × *g*, 4°C, and then washed two times. The final concentration was adjusted to 1 × 10^7^ cells/ml. Then, 100 μl of suspended cells were stained with KO525-conjugated anti-mouse CD45 (Biolegend, 103137, United States), FITC-conjugated anti-mouse CD11b (Biolegend, 101206, United States), and PE-conjugated anti-mouse F4/80 antibody (Biolegend, 123110, United States). The prepared samples were measured and analyzed using Accuri™ C6 flow cytometer (BD Bioscience, United States).

### Plasma LPS Assay

The plasma LPS level in each group was examined using a Limulus amebocyte lysate kit (Xiamen Bioendo Technology Co., Ltd., Xiamen, China) according to the manufacturer's instructions. Briefly, the plasma was diluted with endotoxin-free water (1:4), then 50 μl of diluted plasma was put into each well in a 96-well plate. At the initial time point, 50 μl of the Limulus amebocyte lysate reagent was added to each well. The plate was incubated at 37°C for 30 min. Then, 100 μl of chromogenic substrate warmed to 37°C were added to each well, and the incubation was extended for an additional 6 min at 37°C. Finally, the reaction was stopped by adding 100 μl of 25% solution of glacial acetic acid. Optical density at 545 nm was measured with a microplate reader (Thermo Scientific, United States).

### Immunofluorescence

To further determine the impacts of the dietary ALA-rich FO on inflammatory cells, the Mψs in aortic sinuses were separately detected by immunofluorescence. The frozen sections were taken out from the refrigerator, drying at room temperature for 10 min. After washing with PBS 3 times (3 min/each time), the sections were immersed in paraformaldehyde for 10 min. To minimize the scope of the subsequent incubation of the antibody and to incubate the antibody effectively, we circled the tissue with a histochemical oil pen. After soaking in PBS for 10 min, the sections were blocked with 2% albumin from bovine serum albumin (BSA) for 1 h. Then, the sections were probed with a rat anti-mouse F4/80 (1:250 dilution, Abcam, ab6640, United States) overnight at 4°C. Then samples were incubated with secondary antibody fluorescein (FITC)-conjugated goat anti-rat IgG (H+L) (1:500 dilution, Proteintech, SA00003-11, United States) for 1 h at room temperature, dropping the mounting tablets containing 4',6-diamidino-2-phenylindole (DAPI) (ZSGB-BIO, ZLI-9557, China) chromogenic agent for mounting, observing, and collecting pictures. Images were captured with an Olympus BX51 microscope (Aomori Olympus Co., Ltd., Japan). For quantification, the total numbers of positive cells of each aorta section were determined using the Image J 1.8.0 software. Observers were blinded to the experimental groups.

### Gut Microbiota Analysis

The fecal microbial 16S rRNA gene sequencing and analysis were investigated as the previous studies (29). The mice in each group were transferred to fresh and sterilized cages after 12 weeks of feeding. The fresh feces of each group were individually collected and were immediately frozen into liquid nitrogen, then finally stored at −80°C until the DNA extraction. Cetyltrimethylammonium bromide (CTAB) method was used to extract the genomic DNA of samples, and then the purity and concentration of the DNA were detected by agarose gel electrophoresis (Details in Supplementary Material).

### Fecal SCFAs Quantification by Gas Chromatography-Mass Spectrometer (GC-MS)

The quantification analysis of fecal SCFAs was performed using an Agilent 7890A, gas chromatography coupled with Agilent 5975C mass spectrometric detector (Agilent Technologies, United States) equipped with an HP-5MS column (0.25 × 30 mm, 0.25 μm particle size) (Suzhou Bionovogene Co., Ltd) as described previously (25). Helium was used as a carrier gas at a constant flow rate of 1 ml/min. The initial oven temperature was held at 60°C for 5 min, ramped to 250°C at a rate of 10°C/min, and finally held at this temperature for 5 min. The temperatures of the front inlet, transfer line, and electron impact (EI) ion source were set as 280, 250, and 230°C, respectively. Data handling was performed with an Agilent's MSD ChemStation (E.02.00.493, Agilent Technologies, Inc., United States).

### Fecal BAs Quantification by Liquid Chromatograph-MS (LC-MS)

To detect the composition of BAs, 38 standard solutions were first prepared. Stock solutions were prepared using the solvent described in the instructions and the working solution was prepared using methanol through serial dilution. The standard solutions were stocked under −20°C. Then, we prepared the samples: 100 mg fecal sample was collected and was added with 300 μl methanol to precipitate the protein. Samples were oscillated for 1 min and then centrifuged at 4°C for 10 min (12,000 × *g*). The supernatant was concentrated and dried in a vacuum. The residue was dissolved with 100 μl methanol and the supernatant was ready for LC-MS analysis. The UPLC separation was performed on an Acquity UPLC system (Waters, U.K.) that is equipped with an Acquity UPLC^®^ BEH C18 (1.7 μm, 2.1 × 100 mm, Waters) column. The temperature of the column was set at 40°C. The sample injection volume was 5 μl. The eluents consisted of 0.01% formic acid in water (eluent A) and acetonitrile (eluent B). The flow rate was set at 0.25 ml/min. A 38 min elution gradient was performed as follows: 0–4 min, 25% B; 4–9 min, 25–30% B; 9–14 min, 30–36% B; 14–18 min, 36–38% B; 18–24 min, 38–50% B; 24–32 min, 50–75% B; 32–35 min, 75–100% B; and 35–38 min, and 100–25% B. The MS analysis was performed using an AB 4,000 mass spectrometer (AB, United States) equipped with an ESI source in the negative-ion mode that is working in the multiple reaction monitoring (MRM) mode. An ion source voltage of 4.5 kV, a source temperature of 500°C, and a desolvation temperature of 380°C were used. Collision gas and the curtain gas were set at 6 and 30 psi, respectively, while both atomization gas and auxiliary gas were 50 psi.

### Statistical Analysis

The data were conducted with Prism 8.01 (GraphPad Software Inc., CA, United States). The data were expressed as the mean ± SEM. Differences between multiple comparisons using 2-way ANOVA. According to the normal distribution, the differences between the two groups were analyzed by the Student's *t*-test (2-tailed). Correlation analysis was performed using the Spearman method. The *P* < 0.05 was considered statistically significant.

## Results

### Atherosclerotic Pathological Lesion and Routine Parameters in Diverse Groups

To investigate whether dietary ALA-rich FO can improve AS, the aorta, and aortic sinus in diverse groups were pathologically stained and observed. The *en* face aorta analysis was used to reveal an atherosclerotic lesion formation with the aid of oil-red O staining. The result displayed that the high-fat diet (HFD) has significantly increased the plaque area (*P* < 0.001) (Figure 1B). Nevertheless, the formation of atherosclerotic plaque in the MOD/FO group was reduced compared with the MOD group (*P* < 0.05) (Figure 1B). The extent of atherosclerotic development at the *en* face of the aorta was quantified as a percentage of the total aortic area occupied by the oil-red O-stained lipid deposits (Figure 1C). Moreover, the cross-sectional analysis of atherosclerotic development at the aortic sinus revealed a lipid deposition with the aid of the oil-red O staining in atherosclerotic mice (Figure 1B). The component of atherosclerotic development at the aortic sinus was quantified as a percentage of aortic cross-sectional luminal area occupied by oil-red O-stained lipid deposits. There was a significant difference in the area of lipid plaque between the MOD group and CON group (*P* < 0.01) (Figure 1E), and FO treatment significantly inhibited the excessive lipid deposition and prevented plaque progression (*P* < 0.01) (Figure 1E).

Moreover, Masson's trichrome staining showed atherosclerotic plaques (red) and fibrous fatty plaques (blue) in the aortic sinus (Figure 1B). The component of fibrous fatty plaques at the aortic sinus was quantified as a percentage of the total aortic plaques area occupied by Masson's trichrome-stained fibrous fatty plaques (Figure 1D). A significant difference in fibrous fatty plaques was found between the CON group and MOD group (*P* < 0.01), but there was no significant difference after the intervention of FO (*P* > 0.05) (Figure 1D). Furthermore, the HE staining showed that dietary ALA-rich FO had an amelioration on atherosclerotic plaque (Figure 1B). Taken together, these results indicated that the treatment of dietary ALA-rich FO had the anti-atherogenic property in *ApoE*^−/−^ mice.

We further detected alterations in basic indicators among different groups. At the initiation of the study, there was no significance in body weights (BWs) and in food intake among the 3 groups (Figures 1F,G). At the week 5–6 of HFD, BWs of mice in the MOD group have gained significantly higher than CON group, yet the weights began to decrease after week 10 (all *P* < 0.05), which may be due to a reduced caloric absorption capacity in the progression of the disease. At week 12, BWs in the MOD/FO group were elevated compared to the MOD group (*P* < 0.01) (Figure 1F). This phenomenon suggested that FO intervention has delayed the symptoms of late weight loss in AS. In terms of food intake, the average intake of mice in each group was decreased during the intervention period, but without the significant difference (Figure 1G), suggesting that the effects of FO on BWs were not attributed to the influence of the energy intake. Meanwhile, we detected the levels of plasma TBA. Compared to the CON group, increases in plasma TBA (*P* < 0.05) (Figure 1H) were observed in mice with the MOD group. Intriguingly, the ALA-rich FO intervention has reduced the abnormal TBA (*P* < 0.05) (Figure 1H). This result indicated that the intervention of dietary ALA-rich FO-alleviated AS might be associated with the TBA reduction.

### Dietary ALA-Rich FO Altered the Levels of Plasma/Aortic Inflammatory Cytokines in AS

The anti-inflammatory effect of dietary ALA-rich FO has been demonstrated in different metabolic diseases such as polycystic ovarian syndrome (PCOS) (24) and alcoholic liver disease (ALD) (23). Studies have shown that inflammation is closely related to AS (30). Thus, we further analyzed the influence of FO on inflammation in atherosclerotic mice (Table 1). The results showed that plasma levels of pro-inflammatory TNF-α, IL-1β, IL-6, and IL-17A in the MOD group were significantly increased compared to the CON group (all *P* < 0.05). After the dietary ALA-rich FO intervention, the concentrations of TNF-α, IL-1β, and IL-17A (all *P* < 0.05) in plasma were decreased compared with the MOD group. Meanwhile, the anti-inflammatory IL-10 in the MOD group was notably decreased (*P* < 0.001), but has shown no significant difference between the MOD/FO group and MOD group (*P* > 0.05). These results indicated that dietary ALA-rich FO treatment has ameliorated the systemic inflammation in *ApoE*^−/−^ mice with AS.

**Table 1:** Determination of plasma and aortic inflammatory cytokine levels and plasma lipopolysaccharide (LPS) in diverse groups.

**Inflammatory indicators**	**Groups**		
	**CON**	**MOD**	**MOD/FO**
Aortic TNF-α (ng/mL)	10.97 ± 1.78	39.89 ± 4.35***	33.49 ± 1.96^#^
Aortic IL-1β (ng/mL)	16.27 ± 2.28	21.83 ± 1.19**	18.56 ± 1.13^##^
Aortic IL-10 (ng/mL)	55.19 ± 6.36	48.04 ± 1.91*	52.30 ± 4.09
Aortic IL-6 (ng/mL)	12.28 ± 1.90	15.02 ± 0.50*	13.16 ± 1.43^#^
Aortic IL-17A (ng/mL)	7.55 ± 2.14	15.77 ± 2.15***	12.60 ± 2.46
Aortic MCP-1 (ng/mL)	0.30 ± 0.03	0.35 ± 0.05	0.34 ± 0.04
Plasma TNF-α (ng/mL)	6.50 ± 1.40	10.02 ± 0.97**	8.16 ± 1.46^#^
Plasma IL-1β (ng/mL)	5.22 ± 1.13	11.09 ± 1.73***	6.92 ± 2.01^##^
Plasma IL-10 (ng/mL)	44.15 ± 5.74	3.16 ± 0.29***	3.25 ± 1.15
Plasma IL-6 (ng/mL)	8.76 ± 1.77	12.80 ± 2.09*	12.67 ± 1.38
Plasma IL-17A (ng/mL)	5.00 ± 1.42	9.22 ± 1.53**	7.02 ± 0.84^#^
Plasma MCP-1 (ng/mL)	0.34 ± 0.06	0.38 ± 0.03	0.38 ± 0.03
Plasma LPS (EU/mL)	0.39 ± 0.02	0.44 ± 0.04*	0.38 ± 0.03^#^

*Values are means ± SEM*.

* *P < 0.05*,

** *P < 0.01*,

*** *P < 0.001, CON group vs. MOD group*.

# *P < 0.05*,

## *P < 0.01, MOD group vs. MOD/FO group*.

In addition, we measured the levels of *in situ* aortic inflammatory TNF-α, IL-1β, IL-6 IL-17A, monocyte chemoattractant protein (MCP)-1, and IL-10 to evaluate the effects of dietary ALA-rich FO on plaque inflammation in atherosclerotic mice (Table 1). Similar to the above systemic inflammation, the HFD increased the levels of TNF-α, IL-1β, IL-6, and IL-17A (all *P* < 0.05) in aortic tissues, compared to the CON group. Importantly, these levels were remarkably decreased in MOD/FO group (all *P* < 0.05), except the IL-17A (*P* > 0.05). The IL-10 in the aorta in the MOD group showed a significant decrease compared with the CON group (*P* < 0.05). However, there was no difference in IL-10 between MOD/FO and MOD groups. These results suggested that dietary ALA-rich FO treatment has ameliorated the aortic inflammation in atherosclerotic *ApoE*^−/−^ mice.

### Dietary ALA-Rich FO Inhibited Inflammatory Mψs in AS

**Figure 2:**
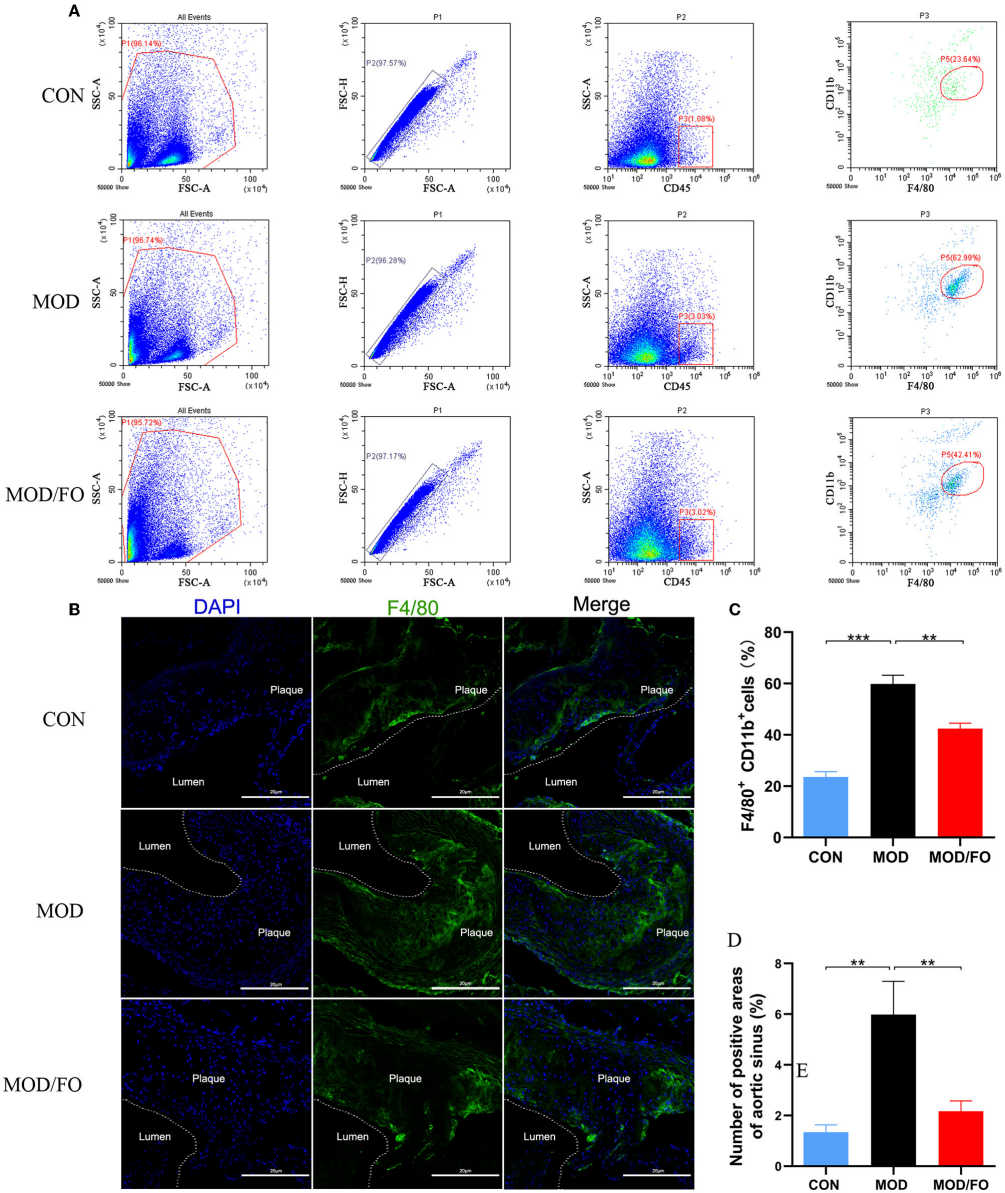
ALA-rich FO inhibited inflammatory Mψ in atherosclerosis by flow cytometry and Immunofluorescence. After obtaining cell suspension from the aortic roots of atherosclerotic *ApoE*^−/−^ mice, the Mψ stained by CD45^+^, F4/80^+^, and CD11b^+^ were detected by flow cytometry. **(A)** Detection of Mψ by KO525 conjugated CD45^+^, PE conjugated F4/80^+^ antibody, and FITC conjugated CD11b^+^ antibody. **(B)** F4/80^+^ cells in immunofluorescence image. In flow cytometry, quantitative analysis as F4/80^+^ CD11b^+^ cells **(C)**. In immunofluorescence assay, quantitative analysis as F4/80^+^ cells **(D)**. Values are given as mean ± SEM. **P < 0.01, ***P < 0.001.

Due to the crucial role of Mψs in the inflammation of AS (31), we measured the Mψs in the orthotopic aortic tissues in diverse groups. After cell suspension from the aortic roots of atherosclerotic *ApoE*^−/−^ mice, the Mψs stained by KO525-conjugated anti-mouse CD45, FITC-conjugated anti-mouse CD11b, and PE-conjugated anti-mouse F4/80 antibody were detected by flow cytometry. We found that Mψs in the MOD group were higher than those in the CON group (*P* < 0.001) (Figures 2A,C). Intriguingly, the proportions of F4/80^+^ CD11b^+^ cells (*P* < 0.01) (Figure 2C) were conversely lower in FO treatment. To further analyze the effects of FO on Mψs in the orthotopic aortic tissues, aortic F4/80^+^ Mψs were measured by immunofluorescence. The aortic plaque inflammatory Mψs were increased after HFD feeding (*P* < 0.01) (Figures 2B,D). Importantly, the dietary ALA-rich FO intervention could dramatically attenuate these abnormal Mψs in the atherosclerotic lesion (*P* < 0.01) (Figures 2B,D).

### Dietary ALA-Rich FO Reduced Plasma LPS Levels

The lipopolysaccharide (LPS), derived from intestinal Gram-negative bacteria, has been widely thought to contribute to the chronic inflammation in diverse metabolic diseases *via* translocation from the gut barrier and activating an inflammatory cascade in Mψ by binding the TLR-4 (18). Thus, we further tested the influence of FO intervention on plasma LPS of AS. The LPS in plasma was significantly increased in the MOD group (*P* < 0.05), compared to the CON group. This elevated LPS in plasma was significantly decreased after the FO intervention (*P* < 0.05) (Table 1), demonstrating that dietary ALA-rich FO possessed the ability to inhibit the LPS generation, lowering the intestinal permeability, as well as stabilizing the intestinal mucosal barrier.

### Dietary ALA-Rich FO-Restored Gut Dysbiosis in AS

The above findings indicated that in AS, an elevated plasma LPS derived from intestinal pathogenic Gram-negative bacteria could be attenuated by a dietary ALA-rich FO treatment, which was closely related to gut dysbiosis. Alterations of gut microbiota have been increasingly considered to play a critical role in the pathogenesis of AS (15, 32, 33). Thus, we further investigated the changes of intestinal flora with the FO intervention. The fecal samples were analyzed by 16S rRNA high throughput sequencing, and the raw reads of gut microbiota in all groups were submitted in National Center for Biotechnology Information's Sequence Read Archive (NCBI SRA) with an accession number PRJNA624814.

**Figure 3:**
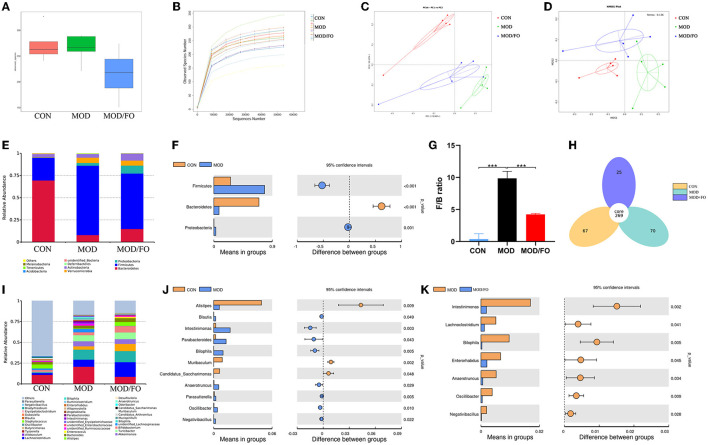
Gut microbial community in fecal samples of different groups. **(A)** Observed-species index. **(B)** Rarefaction Curve. **(C)** PCoA analysis. **(D)** NDMS analysis. **(E–G)** The phylum level. **(H)** Venn diagram. **(I–K)** The genus level. Values are given as mean ± SEM. ***P < 0.001.

As an alpha-diversity, the observed-species' index and rarefaction curve were used to analyze the abundance and diversity of the bacterial community. The observed-species index analysis showed that the abundance and diversity of gut microbiota were altered in the MOD group (*P* < 0.05) (Figure 3A), compared with the CON group. There was no significance after the FO intervention (*P* > 0.05) (Figure 3A). The rarefaction curve tended to be flat when the sequence number increased to 10,000, indicating that the amount of sequencing data was reasonable (Figure 3B). The overall bacterial community structure was analyzed by the unweighted UniFrac (PCoA) (Figure 3C) and by the weighted distance matrices (NMDS) (Figure 3D). We found differential clusters in PCoA of gut microbiota between MOD and CON groups, as well as distinct clusters in PCoA after the FO supplementation compared to the MOD group (Figure 3C). Consistently, the NMDS analysis showed similar results (Figure 3D).

The differential gut bacteria of mice in diverse groups were further determined. First, the Venn diagram showed that 269 core species were observed in all groups, whereas 67, 70, or 25 species were specific in CON, MOD, or MOD/FO group (Figure 3H). Second, at the phylum level, *Firmicutes* and *Bacteroidetes* constituted of 2 dominant bacteria in all groups (Figure 3E). The proportions of *Firmicutes* and *Proteobacteria* were significantly increased and *Bacteroidetes* was decreased in the MOD group compared with those in the CON group (all *P* < 0.05) (Figure 3F). The ratio of *Firmicutes*/*Bacteroidetes* (F/B) was increased (*P* < 0.001) (Figure 3G) in the MOD group, which was reversely decreased after the intervention of dietary ALA-rich FO (*P* < 0.001) (Figure 3G). Thus, the ALA-rich FO had a major influence on the F/B ratio under the HFD feeding in the atherosclerotic mice.

To further evaluate the effect of FO on the genus level of the microbial community, we analyzed the top 40 species (Figure 3I). Compared with those in the CON group, the abundance of *Intestinimonas, Bilophila, Anaerotruncus, Oscillibacter, Negativibacillus, Blautia, Parabacteroides, Muribaculum*, and *Parasutterella* in the MOD group were increased (*P* < 0.05) (Figure 3J), whereas *Alistipes* and *Candidatus Saccharimonas* were decreased. However, after the FO administration, the abundances of *Intestinimonas, Bilophila, Anaerotruncus, Oscillibacter*, and *Negativibacillus* were decreased (*P* < 0.05) (Figure 3K). Importantly, the dietary ALA-rich FO reduced the relative abundances of *Lachnoclostridium* and *Enterorhabdus* compared with the MOD group (all *P* < 0.05) (Figure 3K). Collectively, our results indicate that the HFD consumption significantly altered the initial proportion of OTUs at the genus level, mainly including the increased *Intestinimonas, Bilophila, Anaerotruncus, Oscillibacter*, and *Negativibacillus*, as well as the decreased *Alistipes* and *Candidatus_Saccharimonas*. Conversely, the FO supplementation has restored gut dysbiosis by mainly regulating the *Intestinimonas, Bilophila, Anaerotruncus, Oscillibacter*, and *Negativibacillus*.

### Dietary ALA-Rich FO Increased the SCFAs in Fecal Intestinal Metabolites

**Figure 4:**
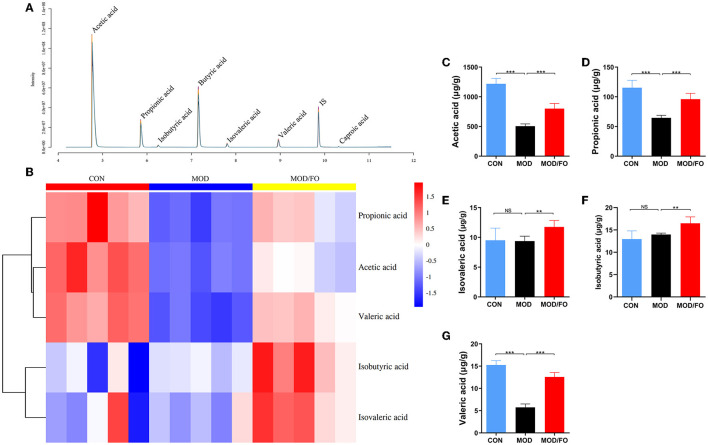
Measurement of the contents of short-chain fatty acids (SCFAs) in fecal intestinal metabolites. **(A)** Chromatogram of mice stool. **(B)** Heat map clustering. **(C)** Determination of levels (μg/g) of Acetic acid, Propionic acid **(D)**, Isovaleric acid **(E)**, Isobutyric acid **(F)**, and Valeric acid **(G)** by gas chromatography-mass spectrometer (GC-MS). Data were presented as mean ± SEM. **P < 0.01, ***P < 0.001, NS, no significance.

The intestinal metabolites' SCFAs have been reported to significantly enhance the intestinal barrier function (34). Due to the effectiveness of FO on restoring gut dysbiosis, we further examined the changes of gut microbiota's metabolites-SCFAs. As shown in the chromatogram (Figure 4A), each SCFA with a single peak could be clearly distinguished, indicating that the method and the data were reliable. The cluster heat map showed the difference in the contents of SCFAs among diverse groups (Figure 4B). The results showed that HFD decreased the amounts of acetic acid, propionic acid, and valeric acid (all *P* < 0.05) (Figures 4C–G) compared to the CON group; whereas the amounts of acetic acid, propionic acid, isovaleric acid, isobutyric acid, and valeric acid were increased after FO treatment, compared with the MOD group (all *P* < 0.05) (Figures 4C–G).

### Dietary ALA-Rich FO Altered the BAs in Fecal Intestinal Metabolites

**Figure 5:**
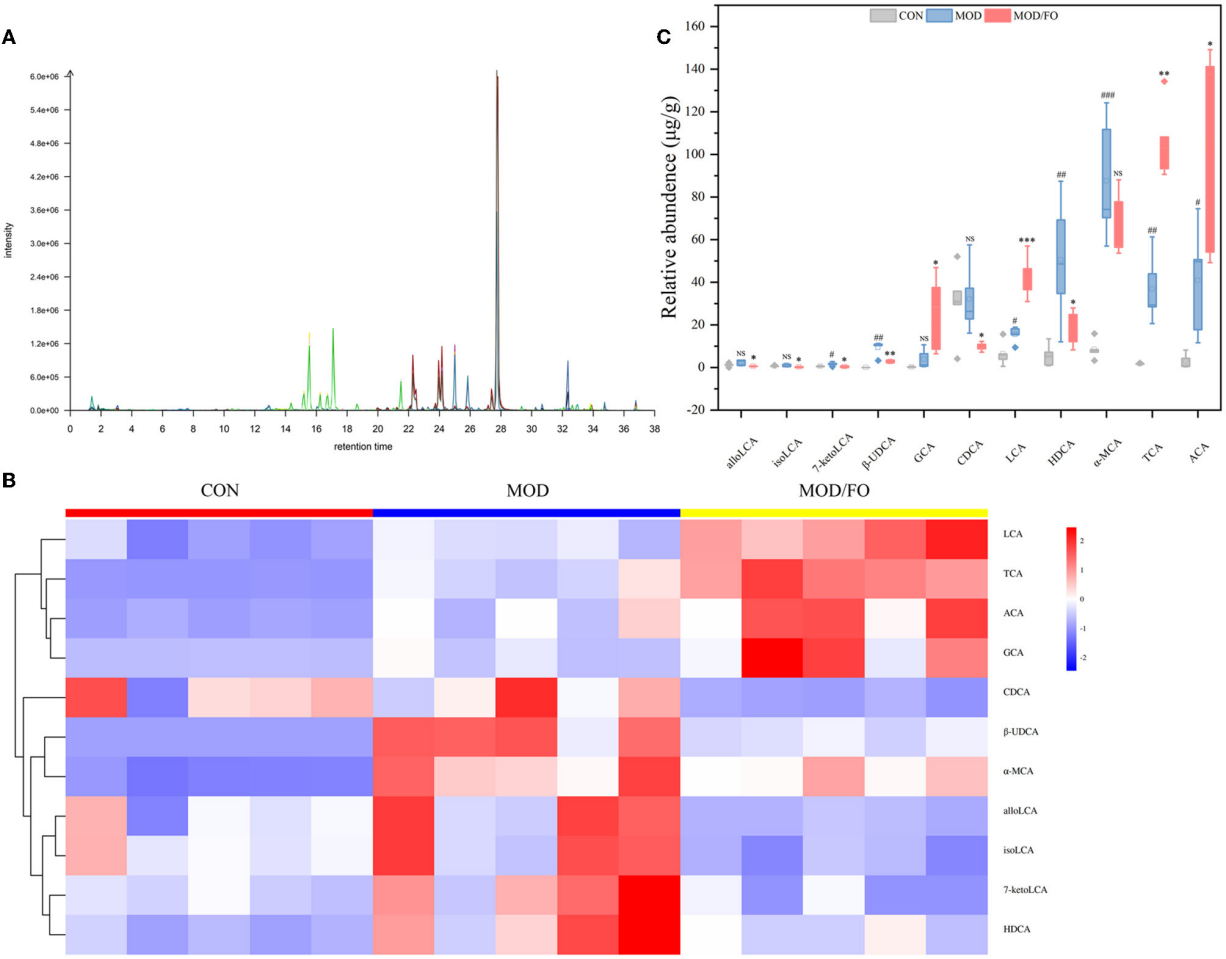
Levels of BAs in fecal intestinal metabolites. **(A)** Chromatogram of mice stool. **(B)** Heat map clustering. **(C)** Determination of levels (μg/g) of allolithocholic acid (alloLCA), isolithocholic acid (isoLCA), 7-ketodeoxycholic acid (7-ketoLCA), β-ursodeoxycholic acid (β-UDCA), chenodeoxycholic acid (CDCA), and hyodeoxycholic acid (HDCA), lithocholic acid (LCA), allocholic acid (ACA), glycocholic acid (GCA), and taurocholic acid (TCA) by Liquid chromatography-mass spectrometer (LC-MS). Data were presented as mean ± SEM. ^#^*P* < 0.05, ^##^*P* < 0.01, ^###^*P* < 0.001, CON group vs. MOD group. *P < 0.05, **P < 0.01, ***P < 0.001, MOD group vs. MOD/FO group. NS, no significance.

Similarly, the BAs, another type of gut microbial metabolite, have been suggested to be involved in the progression of chronic metabolic diseases (35). Thus, we further determined the influence of FO on BAs by LC-MS (Figure 5A). The cluster heat map showed the difference in the contents of BAs among diverse groups (Figure 5B). After FO intervention, allolithocholic acid (alloLCA), isolithocholic acid (isoLCA), 7-ketodeoxycholic acid (7-ketoLCA), β-ursodeoxycholic acid (β-UDCA), chenodeoxycholic acid (CDCA), and hyodeoxycholic acid (HDCA) was reduced (all *P* < 0.05) (Figure 5C), as well as lithocholic acid (LCA), allocholic acid (ACA), glycocholic acid (GCA), and taurocholic acid (TCA) showed an increase (all *P* < 0.05) (Figure 5C), revealing that dietary FO could modulate the microbial BAs metabolism in AS.

### Correlation Analysis Among Gut Microbiota, Intestinal Metabolites, Inflammation

**Figure 6:**
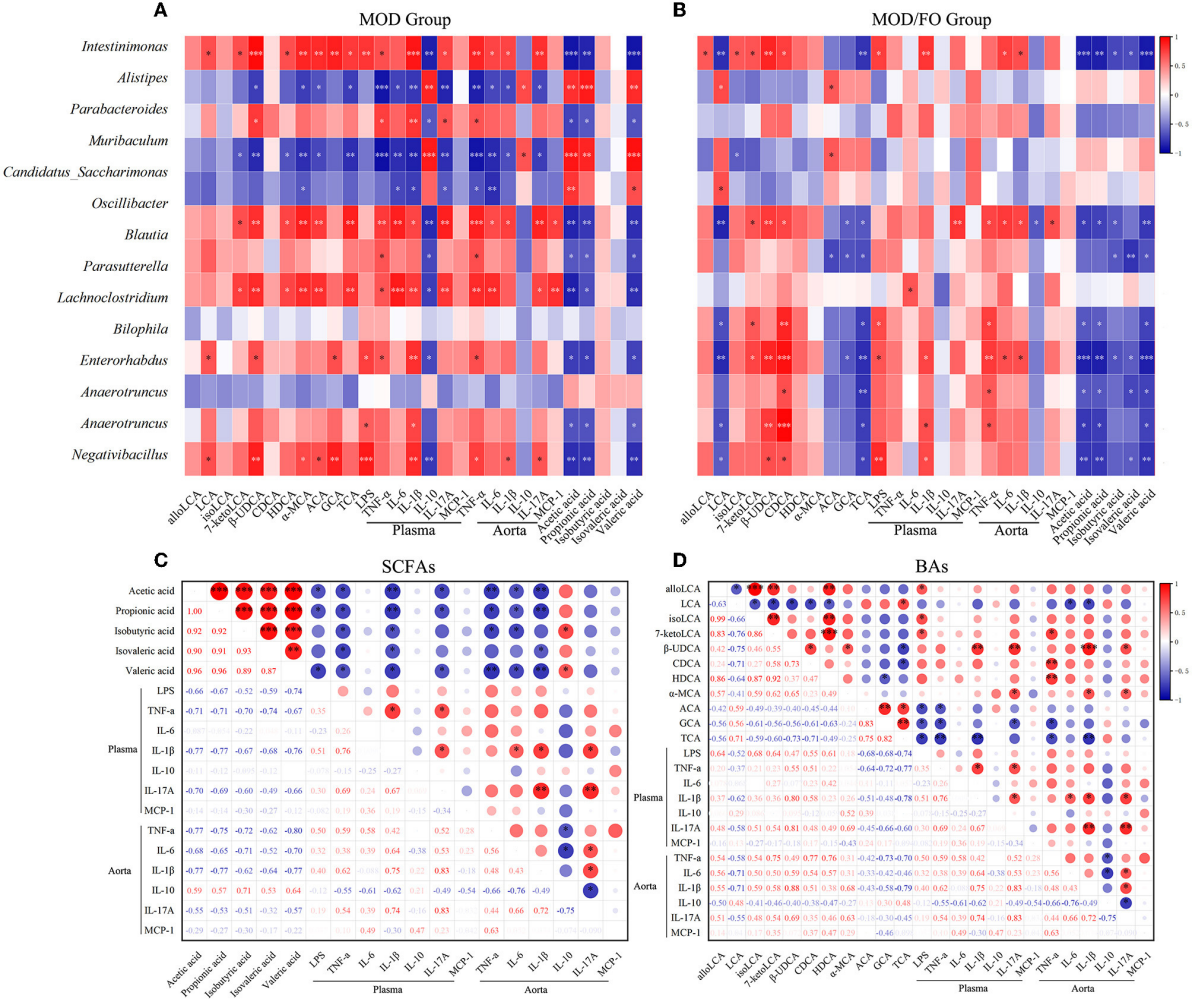
Correlation analysis among bacteria, lipopolysaccharide (LPS), short-chain fatty acids (SCFAs), bile acids (BAs), and inflammatory cytokines. **(A)** Correlation among the differential bacteria with metabolites (SCFAs and BAs), LPS, and plasma/aortic inflammatory indicators in MOD group. **(B)** Correlation among the differential bacteria with metabolites (SCFAs and BAs), LPS, and plasma/aortic inflammatory indicators in MOD/FO group. **(C)** Correlation among SCFAs with LPS and plasma/aortic inflammatory indicators. **(D)** Correlation among BAs with LPS and plasma/aortic inflammatory indicators. *P < 0.05, **P < 0.01, ***P < 0.001. Red and blue represent the positive or negative correlation.

Moreover, we performed a correlation analysis among the differential bacteria, inflammation, LPS, SCFAs, and BAs in AS (Figure 6). The differential bacteria in the MOD group include *Intestinimonas, Bilophila, Anaerotruncus, Oscillibacter, Negativibacillus, Blautia, Parabacteroides, Muribaculum, Parasutterella, Alistipes*, and *Candidatus_Saccharimonas* showed close correlations with inflammation (Figure 6A). Interestingly, the reduced bacteria after the FO intervention involving the *Intestinimonas, Bilophila, Anaerotruncus, Oscillibacter, Negativibacillus, Lachnoclostridium*, and *Enterorhabdus* were positively correlated with pro-inflammatory cytokines and were negatively correlated with anti-inflammatory IL-10 (Figure 6B). In addition, the above downregulated bacteria after the FO intervention were negatively correlated with SCFAs, LCA, ACA, GCA, and TCA, and positively correlated with alloLCA, isoLCA, 7-ketolca, β-UDCA, CDCA, HDCA, and α-MCA (Figure 6). Subsequently, acetic acid, propionic acid, isovaleric acid, isobutyric acid, and valeric acid mainly showed a negative correlation with pro-inflammatory indicators and a positive association with anti-inflammatory IL-10 (Figure 6C). The LCA, ACA, GCA, and TCA were negatively related with inflammatory indicators, whereas other BAs metabolites alloLCA, isoLCA, 7-ketolca, β-UDCA, CDCA, HDCA, and α-MCA were positively correlated with inflammation (Figure 6D). Taken together, the critical differential species and SCFAs/Bas metabolites were determined in close and complicated interactions and correlations among gut bacteria, metabolites, and inflammation.

### Determination of Gut Flora Involved in the Amelioration of Dietary ALA-Rich FO on AS by the Treatment of Antibiotic (AB) Cocktail

**Figure 7:**
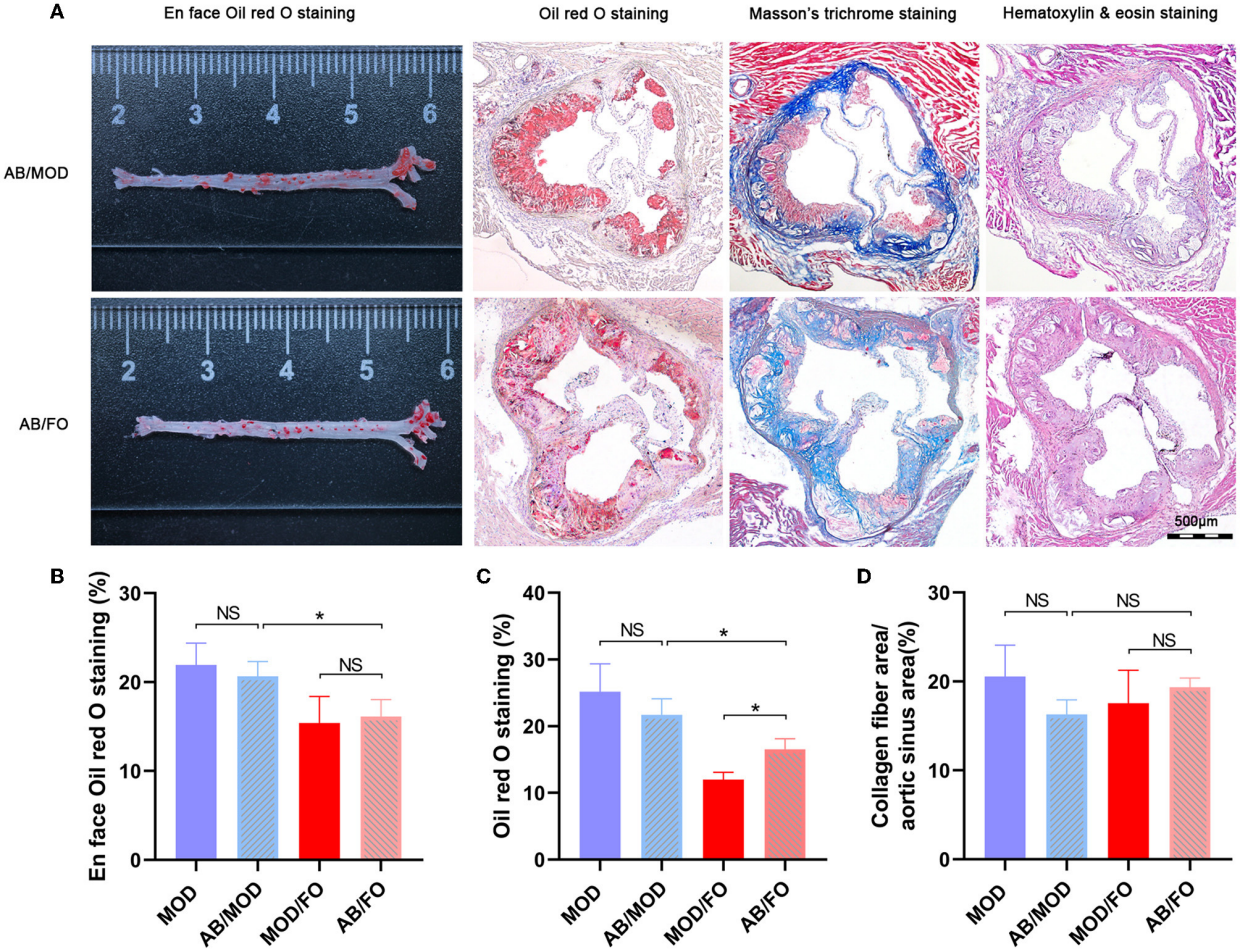
Effects of dietary ALA-rich FO treatment on the pathological lesion in antibiotic (AB)-treated mice. **(A)** Representative sections of the valve area of the aorta and aortic root of the heart were stained with *en* face oil-red O staining, oil-red O staining, Masson's trichrome staining, and Hematoxylin and eosin staining, respectively. Quantitative analysis as lesion area/total area (%) shown in oil-red O staining **(B)**, *en* face oil-red O staining **(C)**, and Masson's trichrome staining **(D)**. *P < 0.05, NS, no significance. Original magnification, ×40. The bar of 500 μm was presented in the right corner of **(A)**. All experiments were performed in triplicates.

In the above studies, we found that the FO administration inhibited the occurrence of inflammation and LPS in AS, related to the disturbance of gut microbiota and microbial metabolites SCFAs/BAs, as well as intestinal barrier function. In this part of the experiment, to further determine whether the amelioration of FO was dependent on the improvement of gut microecology, an AB cocktail treatment was adopted to negatively demonstrate the role of gut microbiota in the FO effectiveness (Figure 7A). Intriguingly, we found that the plaque area in the aortic sinus was higher in AB/FO group than that in the MOD/FO group by oi- red O staining (*P* < 0.05) (Figure 7B), suggesting that the gut dysbiosis rectification contributed to the ALA beneficial effects on AS. Additionally, the area of atherosclerotic plaque in the AB/FO group showed milder symptoms by oil-red O staining (*P* < 0.01) (Figures 7A,B), compared to AB/MOD group, suggesting that ALA-rich FO treatment may partially attenuate the AS through the gut microbiota-independent pathway. The *en* face oil-red O staining and Masson's trichrome staining have shown no significant difference in AB/FO group (*P* > 0.05), compared to AB/MOD group (Figures 7C,D).

**Figure 8:**
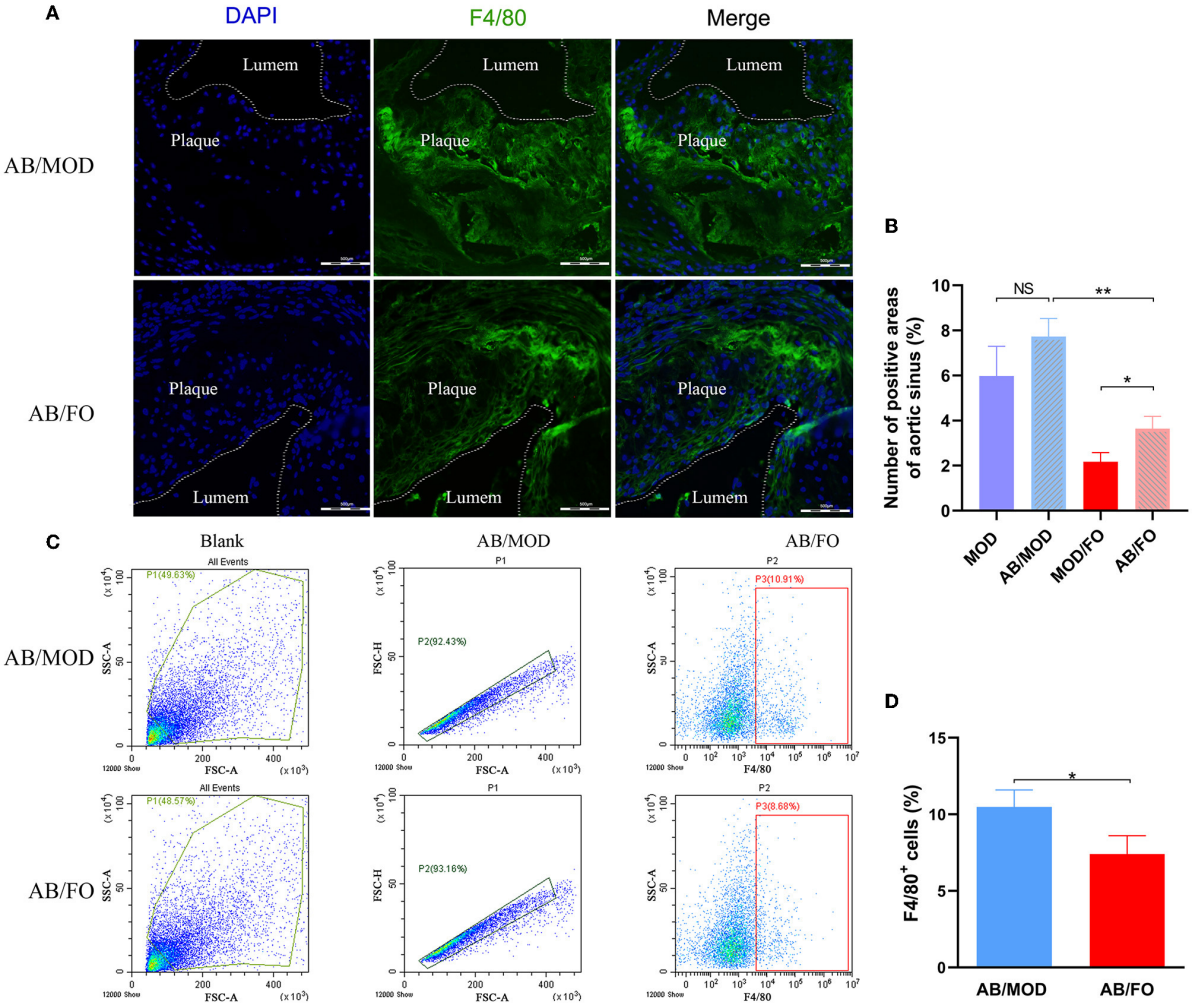
ALA-rich FO inhibited inflammatory Mψ in atherosclerosis by flow cytometry and Immunofluorescence in antibiotic (AB)-treated mice. **(A)** Detection of Mψ by PE conjugated F4/80^+^ antibody. **(B)** F4/80^+^ cells in immunofluorescence image. In flow cytometry, quantitative analysis as F4/80^+^ cells **(C)**. In immunofluorescence assay, quantitative analysis as F4/80^+^ cells **(D)**. Values are given as mean ± SEM. *P < 0.05, **P < 0.01.

Finally, we measured the Mψs by PE-conjugated anti-mouse F4/80 antibody, and the FITC-conjugated anti-mouse TLR4 antibody was detected by flow cytometry in the orthotopic aortic tissues in diverse groups (Figure 8). We found that the aortic Mψs content within aortic lesions was higher in AB/FO group (*P* < 0.05) than that in the MOD/FO group by immunofluorescence, indicating that the modulation of gut microbiota by ALA-rich FO intervention contributed to the anti-inflammation effects on AS. In addition, the aortic Mψs content within the aortic lesions was reduced in the AB/FO group (*P* < 0.01), compared with the AB/MOD group (Figures 8A,B), suggesting that the suppression of the Mψs-mediated inflammation after the ALA administration in AB-treated condition was partially independent on the gut microbiota. The same result occurred in aortic Mψ as detected by flow cytometry (*P* < 0.05) (Figures 8C,D).

## Discussion

In clinical and experimental studies, the consumption of ω-3 PUFAs has exhibited pleiotropic benefits in the control of chronic diseases (36, 37). The dietary ALA-rich FO, a source of plant-derived ω-3 PUFAs, has been widely used as a systemic anti-inflammatory approach in metabolic diseases, such as diabetes and alcoholic fatty liver (23), confirming the reliability of dietary FO. This study mainly explored the protective effects of dietary FO intervention on HFD-induced AS and the underlying gut microbiota-inflammation-artery axis in this effectiveness.

The mice lacking an *apolipoprotein E* are especially vulnerable to a series of complex vascular lesion symptoms under the stimulation of HFD, which were comparable to human lesions (38). In this study, we confirmed that FO could improve the AS pathological injury in atherosclerotic *ApoE*^−/−^ mice with HFD. The FO intervention on the food intake in our study showed a limited influence, which indicated that the effectiveness of FO treatment on AS was not dependent on nutrients absorption and on the efficiency of calorie utilization in the gastrointestinal tract in AS, which was consistent with previous reports (12).

To further reveal the underlying mechanisms of dietary FO amelioration on AS, we assessed the gut microbiota alterations due to a critical role of gut dysbiosis in the pathogenesis of AS (39, 40). In this study, we found that phyla *Bacteriodetes* and *Firmicutes* were kept predominantly in diverse groups, which are paralleled with previous studies (18, 23, 32). An increase in *Firmicutes*/*Bacteriodetes* ratio is closely related to chronic metabolic diseases [(24, 25, 41)]. In this study, the increased ratio of *Firmicutes*/*Bacteriodetes* in AS was rectified by the dietary FO administration, suggesting that FO could markedly modulate the gut microbiota by decreasing the predominant *Firmicutes*/*Bacteriodetes* in phylum level (42).

Moreover, at the genus level, the reduced intestinal bacteria after the FO administration, including *Anaerotruncus, Enterorhabdus, Lachnoclostridium, Negativibacillus*, and *Bilophila*, were Gram-negative bacteria and anaerobic bacteria, which were closely related to the production of LPS (43, 44). In these differential bacteria, the *Anaerotruncus* that was isolated from the blood culture of a 78-year-old woman with nosocomial bacteraemia, is of clinical significance (45). Clavel (46) first isolated the *Enterorhabdus* from patients with colitis and found that it was probably associated with the inflammation of the disease (46). Intriguingly, relevant studies have shown that *Lachnoclostridium* is significantly enriched in tumors and has been identified as a novel bacterial marker for the non-invasive diagnosis of colorectal adenoma (47). Early studies have shown that a high-fat dairy diet in mice will increase bile production, leading to an increase in *Bilophila* abundance (48). Taken together, the improvement of FO intervention on AS may be due to the rectification of gut dysbiosis. In order to determine the crucial role of gut microbiota in the effectiveness of FO on the development of AS, as previously described (28), the AB cocktails were used to eliminate the effect of gut microbiota in *ApoE*^−/−^ mice. After 3 weeks of AB cocktails, gut microflora was found with difficulty by 16S rRNA sequencing, demonstrating that mimic germ-free mice were successfully established. During the subsequent 12 weeks of treatments in diverse groups, a significantly weakened amelioration of dietary FO treatment on AS suggested that intestinal microbiota contributed to the FO effectiveness.

The LPS, derived from pathogenic bacteria in gut dysbiosis, represents a causal link between gut microbiota and low-grade systemic inflammation (49). Numerous studies have demonstrated that andenterogenic endotoxemia-mediated systemic chronic inflammation aggravates the pathogenesis of AS (50). Our data revealed that the LPS in plasma were markedly decreased with FO supplementation, demonstrating that dietary FO intervention may improve the gut permeability, thus enhancing the gut barrier, to decrease the production of LPS translocation to the systemic circulation. A previous study indicated that genetic deletion of *TLR-4* and *Myd88* in *ApoE*^−/−^ mice were associated with a reduced Mψ inflammatory response and aortic lesions (11). Thus, we speculated that the anti-inflammation role of dietary ALA-rich FO on AS in *ApoE*^−/−^ mice might be through the LPS/TLR-4/NF-κB pathway, but the exact evidence needs to be further researched.

The above demonstrated the attenuation of FO intervention on AS through rectifying the gut dysbiosis, enhancing the gut barrier and conversing the LPS translocation. Subsequently, the impacts of dietary FO on LPS mediated-inflammation consequences were further determined. Numerous studies have solidly demonstrated that chronic inflammation is dramatically involved in the development and the rupture of atherosclerotic plaque (51, 52). The Mψs exerted a predominant inflammatory role in AS lesion formation, as well as plaque rupture (53, 54). In the present study, the proportions of F4/80^+^ cells and F4/80^+^ TLR4^+^ cells was significantly decreased with the dietary FO administration, suggesting that the anti-inflammation effect of ALA-rich FO intervention might be mainly due to the inhibition of inflammatory Mψs. We speculated that ALA-rich FO may suppress the pro-inflammatory M1 Mψs against the exacerbated inflammation and, thus, limit the aortic injury. However, the exact molecular mechanism of FO on Mψs polarization needs to be further researched. In parallel, pro-inflammation plasma TNF-α, IL-1β, and IL-17A, as well as the aortic tissues TNF-α, IL-6, and IL-1β were suppressed after the treatment. Also, further correlation analysis discovered that pro-inflammatory cytokines and Mψs were closely related to the above differential intestinal flora, indicating that FO may regulate the gut microbiota for the contribution of inflammation suppression. Additionally, whether other inflammatory-related cells, such as regulatory T cells (Tregs), T helper cell 17 (Th17), and myeloid-derived suppressor cells (MDSCs), are involved in the improvement of FO on AS inflammation still need to be investigated in our subsequent research. Moreover, intriguingly, we found during the AB-treated condition that the FO intervention could also lower the inflammatory levels, suggesting that the anti-inflammation effect of ALA-rich FO may be partially independent of modulation of gut microbiota. Emerging evidence has demonstrated that ω-3 PUFAs, including ALA, EPA, and DHA, can serve as a novel anti-inflammation approach by directly binding these to the G protein-coupled receptor (GPR) 40, GPR120, and GPR119 (12, 27). The interconversions among diverse ω-3 PUFAs and related signaling pathways are further investigated.

In the process of dietary FO administration on rectifying the gut dysbiosis in AS, gut microbiota metabolites SCFAs, tryptophan metabolism, Bas metabolism, and trimethylamine oxide (TMAO) have been reported to play a critical role in the occurrence and the development of chronic metabolic diseases (55). The SCFAs, a type of critical gut microbiota metabolites, as a link between microbiota and host homeostasis, play an important role in the regulation of inflammation and intestinal barrier function (56). However, the effect of dietary FO intervention on SCFAs in AS was only few as known previously. In this study, the increased levels of acetic acid, propionic acid, isobutyric acid, isovaleric acid, and valeric acid after dietary FO administration were negatively correlated with inflammatory indicators, indicating the beneficial effects of SCFAs in the attenuation of FO on AS. Moreover, the anti-inflammatory and immunomodulatory effects of SCFAs might be due to the activation of specific cell receptors, such as the GPR41, GPR43, and major intestinal receptors GPR109a. Additionally, the intracellular target of SCFAs *via* inhibiting the activity of histone deacetylase (HDACs) is involved in the regulation of expression of genes promoting pathogenesis in many diseases (57). Besides, the SCFAs promote the increase of intestinal TJP (tight junction protein) through intestinal mucosal receptors of monocarboxylate transporter 1 (MCT-1) and sodium-coupled monocarboxylate transporter 1 (SMCT-1), to block the LPS translocation into the blood circulation and to ultimately suppress the systemic inflammation (19). The accurate mechanisms of these different SCFAs metabolites *via* binding to multiple specific corresponding sensors in AS, with or without dietary FO treatment, still need to be separately determined.

Apart from SCFAs, the BAs' metabolites play an essential role in integrating multiple homeostatic functions in the liver and gastrointestinal tract (58). However, the impact of dietary FO intervention on BAs in AS was little known previously. Interestingly, a significant decrease of the plasma TBA indicated that the anti-AS effect of FO may be associated with the regulation of the BA biosynthesis. Furthermore, a series of differential BAs' metabolites, including decreasing alloLCA, isoLCA, 7-ketoLCA, β-UDCA, CDCA, and HDCA, as well as up-regulating LCA, ACA, GCA, and TCA, were found with the dietary FO administration, showing that the ALA-rich FO had a capacity of regulating the gut microbiota-associated BAs' metabolites for the potential protection against AS. Further correlation analysis also demonstrated that these differential BAs' metabolites were markedly related to atherosclerotic inflammation. Accumulating studies have revealed that BA-sensing receptors, including farnesoid X receptor (FXR) and G protein-coupled BA receptor 1 (TGR5) (59), have mediated the anti-inflammation effects *via* NcoR1-NF-κB/NLRP3 and cAMP-PKA-NF-κB/NLRP3 pathways, respectively (60, 61). Thus, the exploration of the underlying mechanisms of these differential BAs' metabolites *via* binding to specific receptors in AS, with or without dietary FO treatment, might contribute to the understanding of BAs' feedback regulation in AS.

## Conclusion

This study highlighted that the dietary ALA-rich FO mainly ameliorated the HFD-induced AS *via* gut microbiota-inflammation-artery axis in *ApoE*^−/−^ mice, which potentially served as the inexpensive interventions for the prevention and treatment of the disease.

## Data Availability Statement

The datasets presented in this study can be found in online repositories. The names of the repository/repositories and accession number(s) can be found below: https://www.ncbi.nlm.nih.gov/, PRJNA624814.

## Ethics Statement

This animal experiment has been approved by Ningxia Medical University Ethics Committee (No. 2019-137).

## Author Contributions

HW, XZ, and YiL: conceptualization and validation. YiL, ZY, YuL, TW, YaL, ZB, YR, HM, TB, HL, RW, LY, NY, XZ, and RY: methodology and investigation. YiL and ZY: software and formal analysis. HW: resources and project administration. YiL, ZY, YuL, and TW: data curation. YiL, ZY, and HW: writing—original draft preparation. HW and YiL: writing—review and editing. YiL: visualization. XZ and HW: supervision. HW, XZ, and SJ: funding acquisition. All authors contributed to the article and approved the submitted version.

## Funding

This research was funded by National Natural Science Foundation of China, Grant Number: 82160691, Ningxia Natural Science Foundation, China, Grant Numbers: 2021AAC05010, 2020AAC03002, and 2020AAC03132, the Key Research and Development Projects of Ningxia, China, Grant Number: 2018BEG02006, the Research Project of Ningxia Medical University, Grant Number: XT2018007, and Orthopedic Department Group Project of Ningxia Medical University, Grant Numbers: XY201627 and XY201529.

## Conflict of Interest

The authors declare that the research was conducted in the absence of any commercial or financial relationships that could be construed as a potential conflict of interest.

## Publisher's Note

All claims expressed in this article are solely those of the authors and do not necessarily represent those of their affiliated organizations, or those of the publisher, the editors and the reviewers. Any product that may be evaluated in this article, or claim that may be made by its manufacturer, is not guaranteed or endorsed by the publisher.
