# Supplementary material for: Dietary α-Linolenic Acid-Rich Flaxseed Oil Ameliorates High-Fat Diet-Induced Atherosclerosis via Gut Microbiota-Inflammation-Artery Axis in ApoE−/− Mice
Source: Front Cardiovasc Med. 2022 Feb 28;9:830781. doi: 10.3389/fcvm.2022.830781 (PMC8918482; doi:10.3389/fcvm.2022.830781)
Supplement: Supplementary file 1 [file Table_1.docx]

**1. Materials and Methods**

1.1 Histology and morphometry evaluations of atherosclerotic lesions

For en face analysis, aorta from the ascending arch to the iliac bifurcation was detached and cleaned, then opened them longitudinally, pinned flat, and stained with oil-red O. Images were captured with Canon EOS 70D camera.

By placing the heart under a stereo microscope, the distal end of the aortic sinus can be identified and separated by the disappearance of the 3 aortic valve cusps. The aortic sinus samples were embedded in tissue freezing medium optimal cutting temperature compound (O.C.T.) and sectioned into continuous 8 mm thick sections at -20°C. Every 6 sections were stained with oil red O and digitally photographed under magnification (×40).

The frozen section was stained with oil red O, hematoxylin and eosin (H&E) and Masson's trichrome. Whole coloured slides were observed under the Olympus light microscope (Melville, NY) for evaluation of the atherosclerotic plaque.

Oil red O staining: Sections in 4% paraformaldehyde were incubated for 15 min, then immersed in 60% isopropanol for 10 min. And then sections were put into the modified oil red O staining solution and stained for 15 min. 60% isopropyl alcohol was used to remove the staining. After washing with distilled water, Mayer hematoxylin staining was adopted to counterstain the nucleus for 20 s. Finally, after the glycerin gelatin seal, lipid accumulation in atherosclerotic plaques was observed by the microscope.

HE staining: Frozen sections were stained with H&E to evaluate the degree of atherosclerotic plaque.

To assessed fibrous tissue hyperplasia in atherosclerotic plaques, Masson's trichrome staining was performed. In brief, frozen sections were put in 4% paraformaldehyde for 10 min and stained with the Weigert iron hematoxylin for 5-10 min. After that, the tissue sections were differentiated with acidic ethanol differentiation solution. Subsequently, the slides were washed with tap water for 5 min and dyed them blue with Masson's blue solution. After washing them with distilled water, the tissue sections were dyed with Ponceau red magenta staining solution for 5-10 min. Next, the slides were washed with a weak acid working solution phosphomolybdic acid solution for 1-2 min in turn. After washing them with the prepared weak acid working solution for 1 min, the sections were put into aniline blue staining solution for 1-2 min. Then we used a weak acid working solution to wash them for 1 min. 95% ethanol was used to dehydrate the sections quickly, and then anhydrous ethanol was used to dehydrate 3 times (5-10 min/each time). Then xylene was used to transparent them 3 times (1-2 min/each time). Finally, we used neutral gum to seal slides.

Images were analyzed using Image J 1.8.0 software (National Institutes of Health, United States). The lesion area index was calculated as the percentage of aortic lumen area covered by atherosclerotic lesions. Observers were blinded to the experimental groups.

1.2 Gut microbiota analysis

The fecal microbial 16S rRNA gene sequencing and analysis were investigated as previous studies30. Mice in each group were transferred to fresh and sterilized cages after 10 weeks of feeding. The fresh feces of each group were individually collected and immediately frozen into liquid nitrogen, finally stored at -80°C until DNA extraction.

Cetyltrimethylammonium bromide (CTAB) method was used to extract the genomic DNA of samples, and then the purity and concentration of the DNA were detected by agarose gel electrophoresis. An appropriate amount of samples was placed in a centrifuge tube, and samples were diluted to 1 ng/µL with sterile water. The V3 and V4 regions of 16S ribosomal DNA genes were amplified by PCR with barcode-indexed specific primers (341F and 806R). All PCR reactions were carried out in 30 µL reactions with 15 µL of Phusion® High-Fidelity PCR Master Mix (New England Biolabs), 0.2 μM of forward and reverse primers and 10 ng template DNA. Thermal cycling consisted of initial denaturation at 98°C for 1 min, followed by 30 cycles of denaturation at 98°C for 10 s, annealing at 50°C for 30 s, and elongation at 72°C for 30 s. Finally, the samples were performed at 72°C for an additional 5 min.

Products and operate electrophoresis of PCR were separated by 2% agarose gel electrophoresis. PCR products were mixed in equidensity ratios. Then, PCR products were purified with Gene JETTM Gel Extraction Kit (Thermo Scientific). Sequencing libraries were generated using Ion Plus Fragment Library Kit 48 rxns (Thermofisher) following manufacturer's instructions. The library quality was assessed on the Qubit@ 2.0 Fluorometer (Thermo Scientific). The library was sequenced on Ion S5TMXL platform by Novogene Bioinformatics Technology Co. Ltd. (Beijing, China) and 400-450 bp single-end reads were generated eventually.

The single-end reads were assigned to samples based on their unique barcode and truncated by cutting off the barcode and primer sequence. Quality filtering on the raw reads was performed under specific filtering conditions to obtain the high-quality clean reads according to the Cutadapt (V1.9.1) quality-controlled process. Chimera sequences were identified and deleted. Then the clean reads finally were obtained. Sequences analysis was performed by Uparse software (Uparse v7.0.1001), Sequences with ≥97% identity were assigned to the same operational taxonomic units (OTUs). A representative sequence for each OTU was screened.

To make the results of information analysis more accurate and reliable, quality filtering on reads were performed under specific filtering conditions to obtain the high-quality clean reads according to the Cutadapt (V1.9.1) quality controlled process. Based on the valid data, OTUs clustering and species classification analysis were performed. And OTU and species annotation were combined to obtain the basic analysis results of OTUs and taxonomic lineages for each sample. Then, the abundance and diversity index of OTUs were analyzed. Simultaneously, the community structure statistical analysis was performed on the species annotation at various classification levels. Finally, according to the above analysis, a series of cluster analysis based on OTUs and species composition, statistical comparison and analysis of principal co-ordinates analysis (PCoA) and principal components analysis (PCA), canonical correspondence analysis (CCA), and redundancy analysis (RDA) were performed to uncover the differences in species composition between samples and combine environmental factors.
