# Supplementary figures and images for: Dietary α-Linolenic Acid-Rich Flaxseed Oil Ameliorates High-Fat Diet-Induced Atherosclerosis via Gut Microbiota-Inflammation-Artery Axis in ApoE−/− Mice
Source: Front Cardiovasc Med. 2022 Feb 28;9:830781. doi: 10.3389/fcvm.2022.830781 (PMC8918482; doi:10.3389/fcvm.2022.830781)

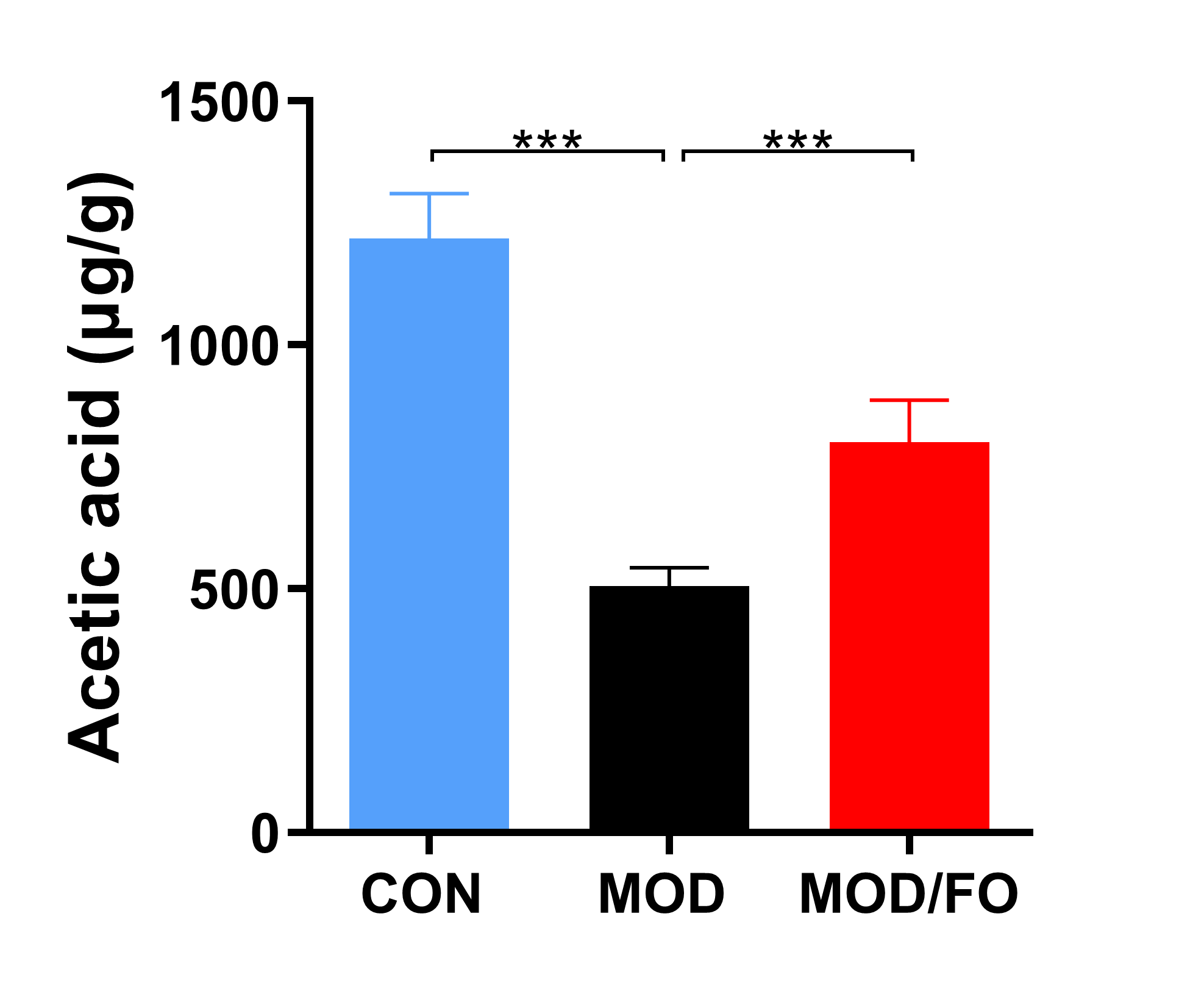

Supplement: Supplementary file 3 [file Data_Sheet_2.ZIP › SCFA/Acetic.acid.tif]

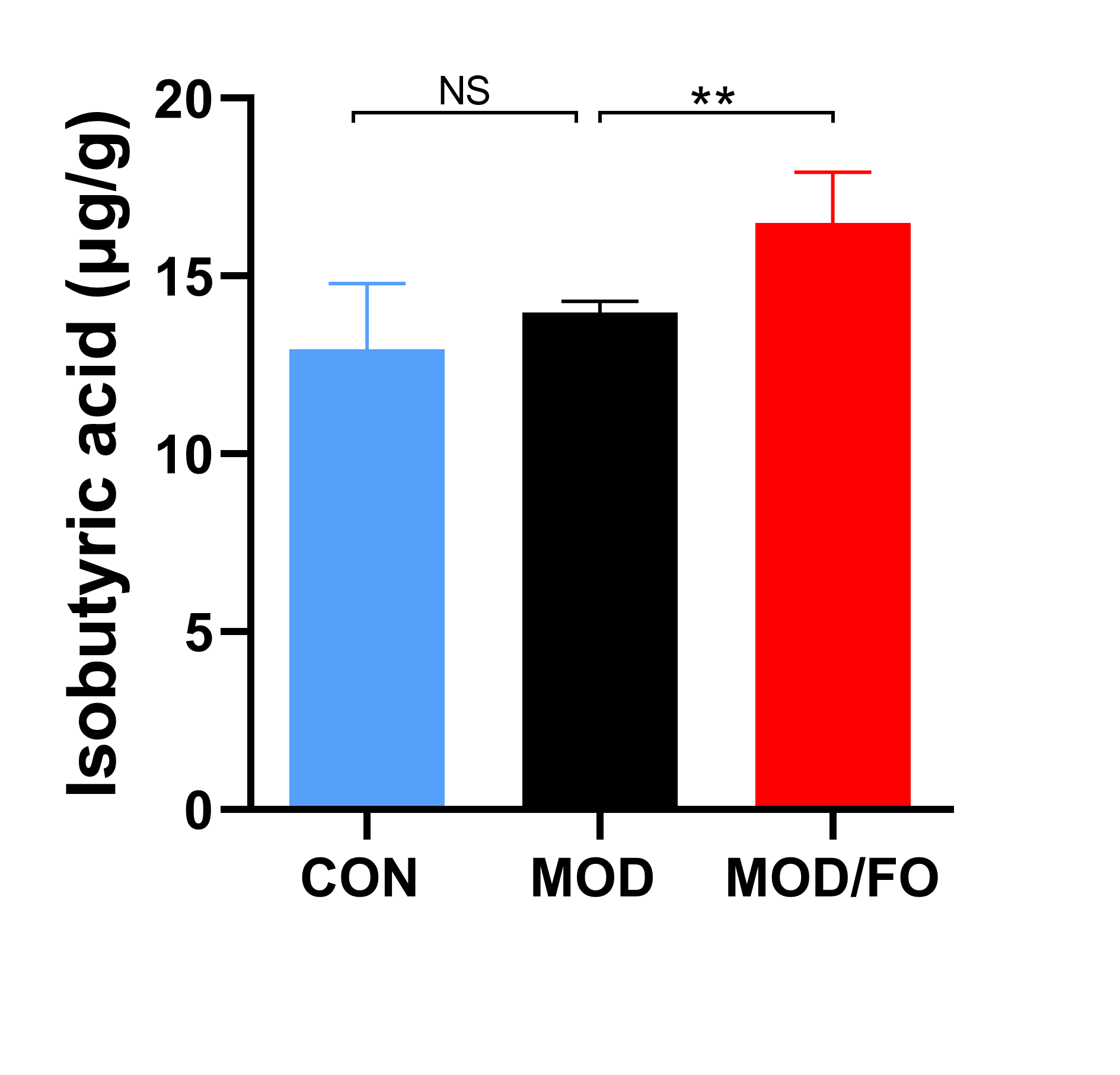

Supplement: Supplementary file 3 [file Data_Sheet_2.ZIP › SCFA/Isobutyric.acid.tif]

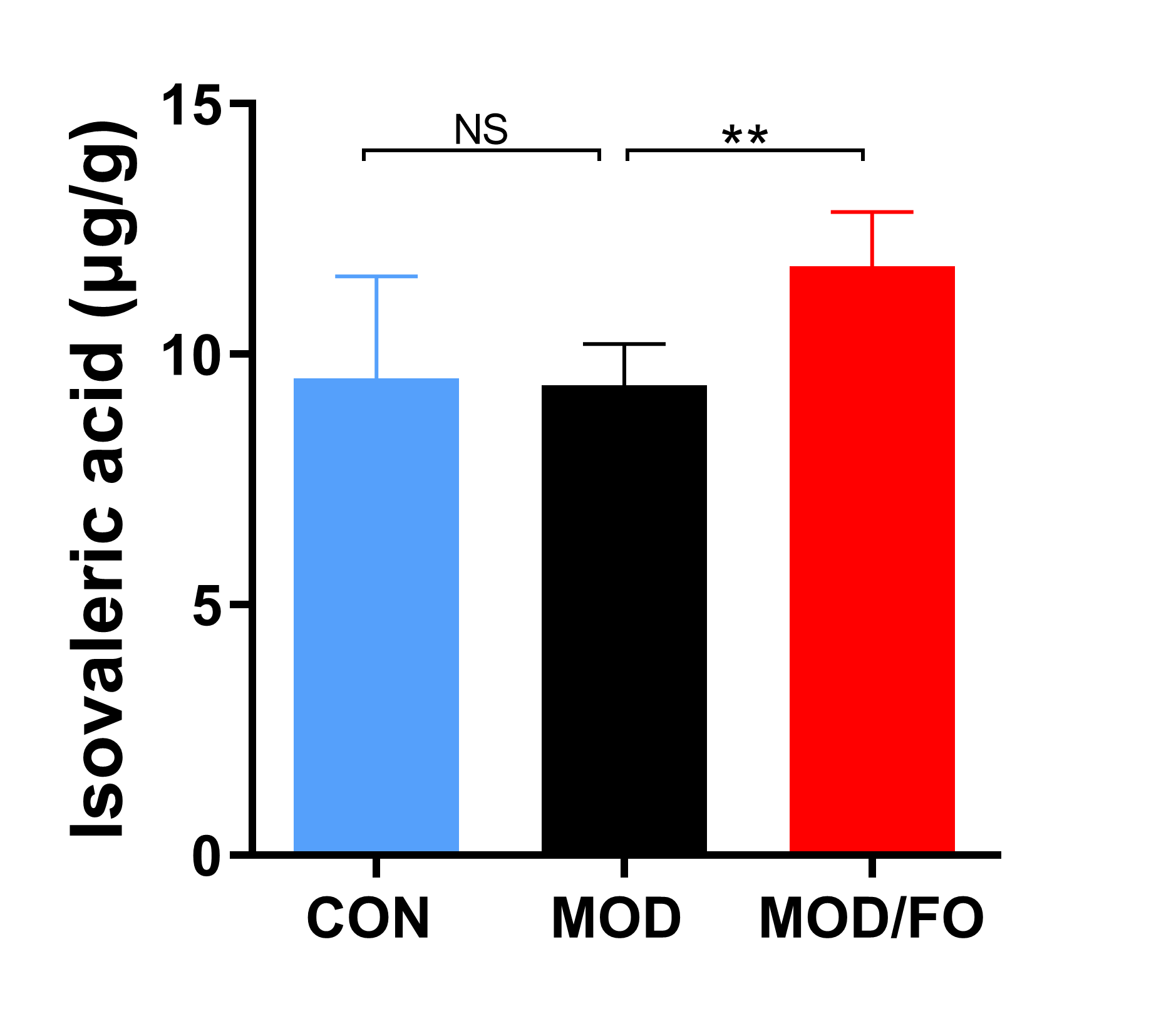

Supplement: Supplementary file 3 [file Data_Sheet_2.ZIP › SCFA/Isovaleric.acid.tif]

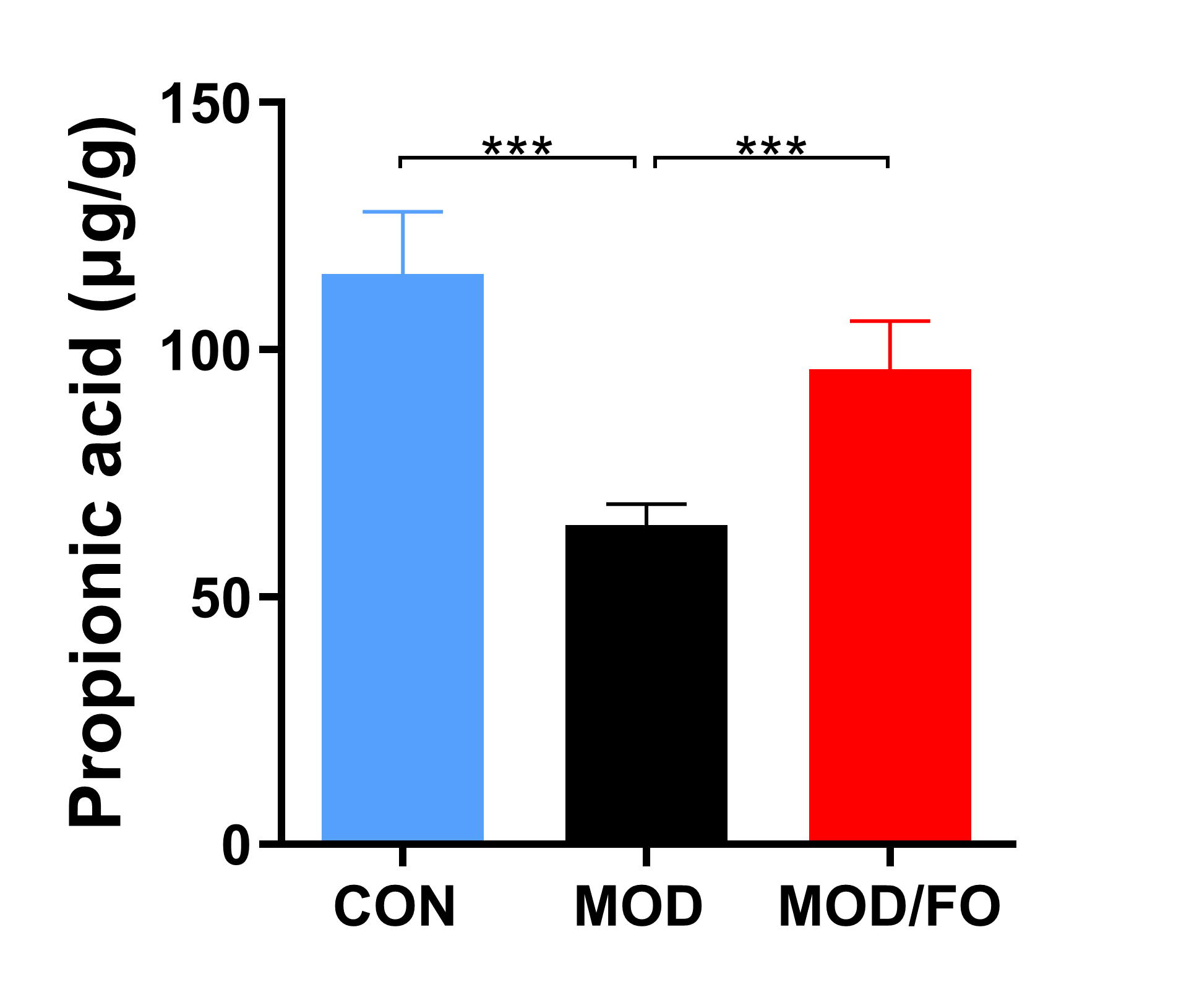

Supplement: Supplementary file 3 [file Data_Sheet_2.ZIP › SCFA/Propionic.acid.tif]

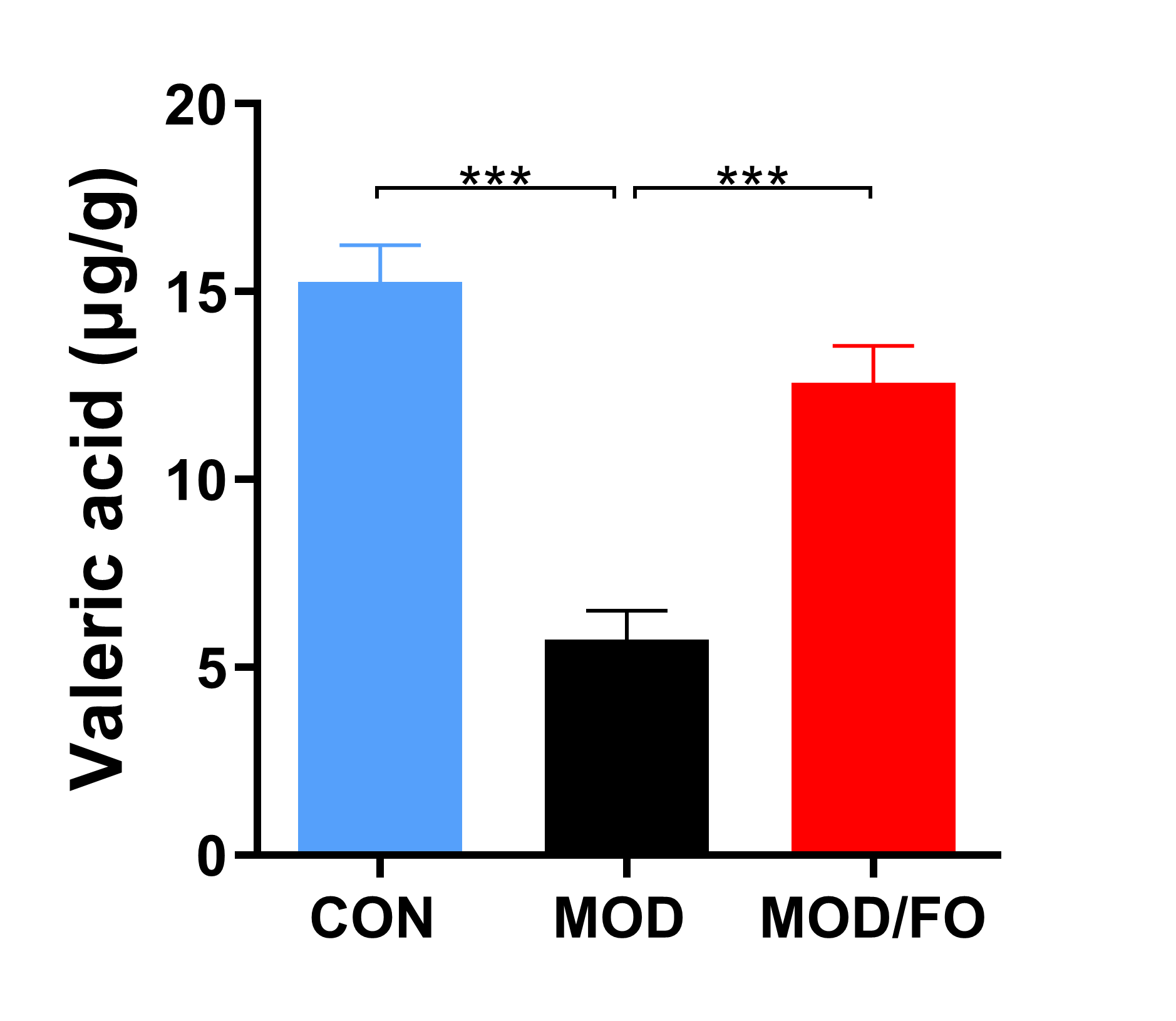

Supplement: Supplementary file 3 [file Data_Sheet_2.ZIP › SCFA/Valeric.acid.tif]

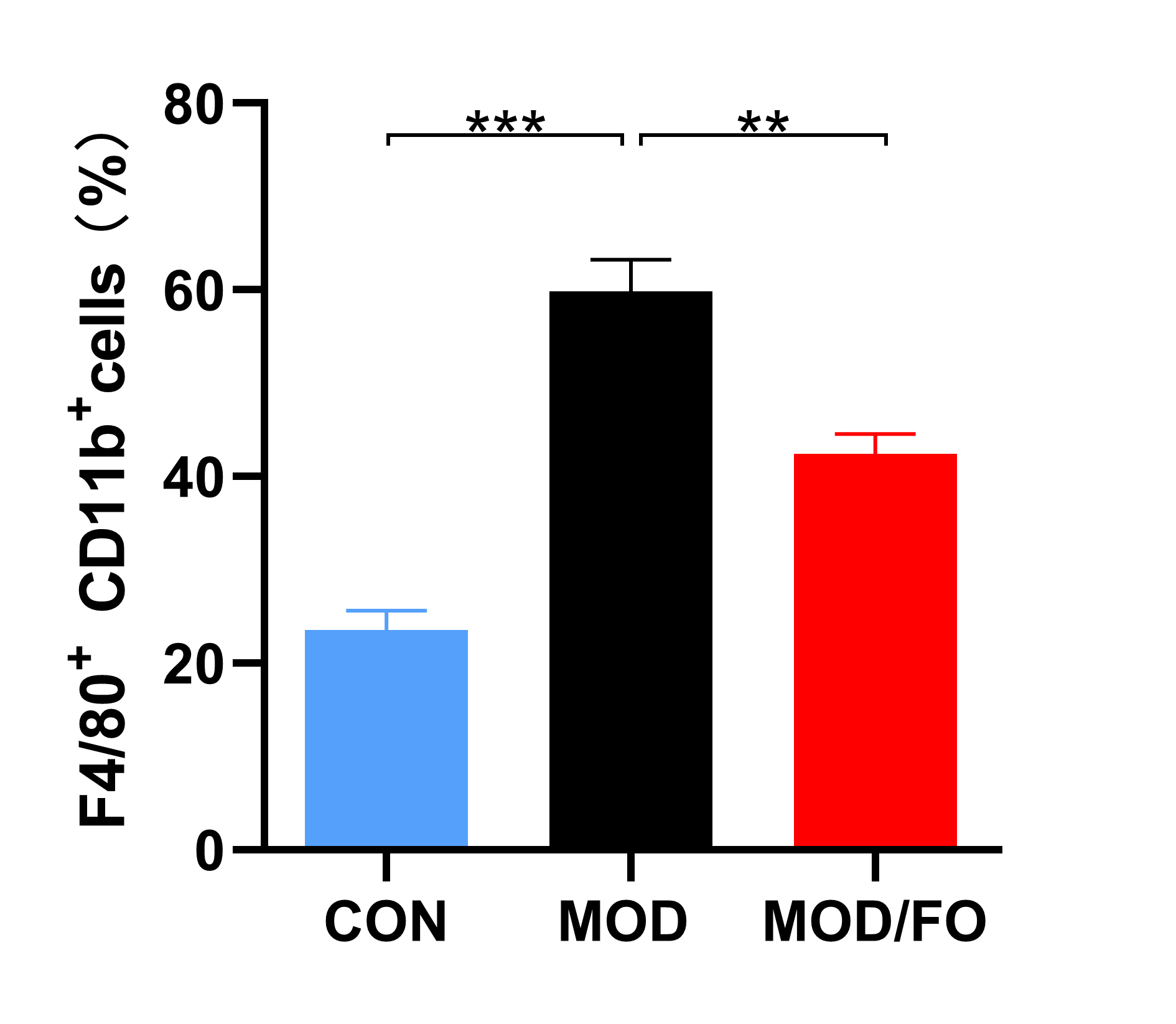

Supplement: Supplementary file 6 [file Data_Sheet_5.ZIP › macrophages/FLOW/flow-f480 CD11b.tif]

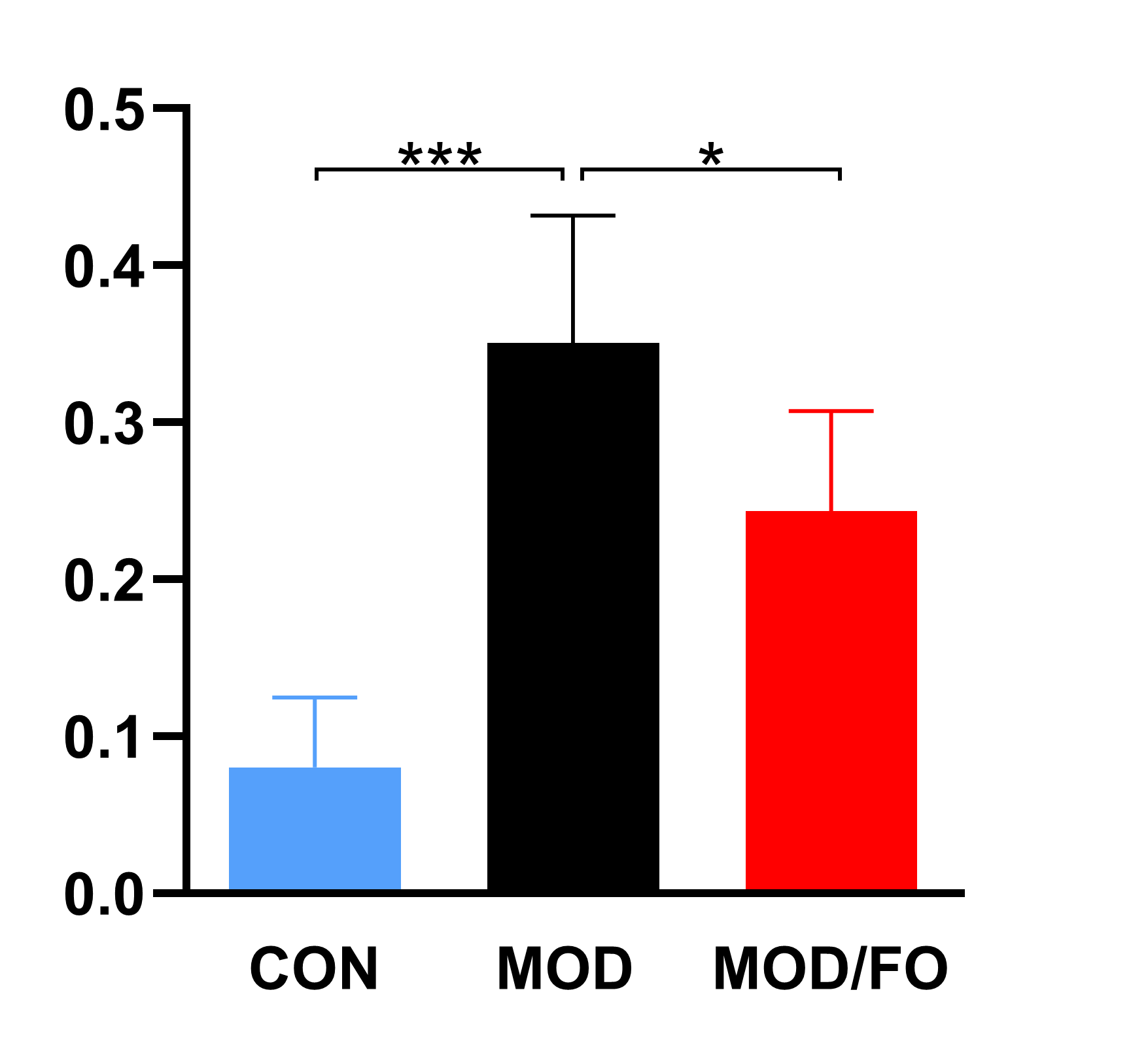

Supplement: Supplementary file 6 [file Data_Sheet_5.ZIP › macrophages/immunofluorescence/immunofluorescence.tif]

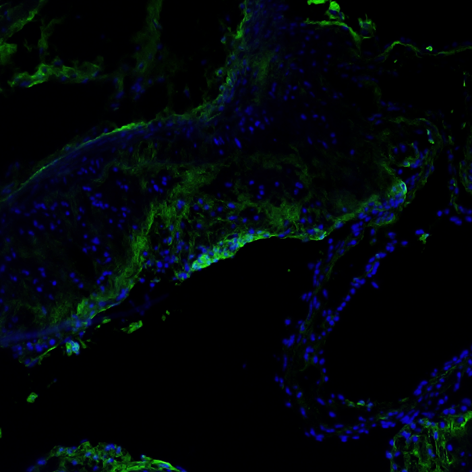

Supplement: Supplementary file 6 [file Data_Sheet_5.ZIP › macrophages/immunofluorescence/original figure/CON 1.tif]

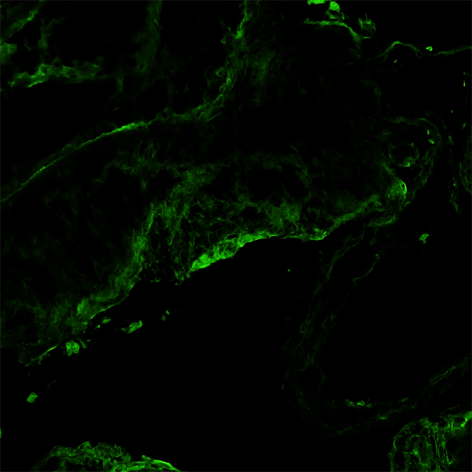

Supplement: Supplementary file 6 [file Data_Sheet_5.ZIP › macrophages/immunofluorescence/original figure/CON 2.tif]

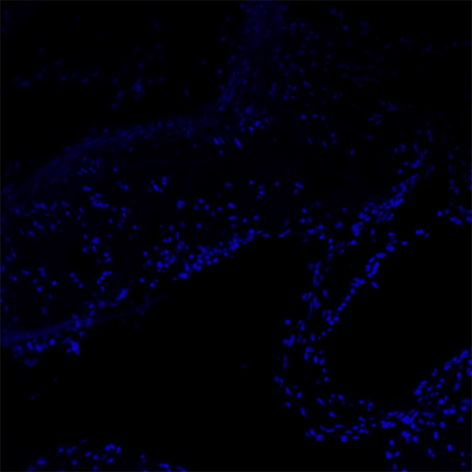

Supplement: Supplementary file 6 [file Data_Sheet_5.ZIP › macrophages/immunofluorescence/original figure/CON 3.tif]

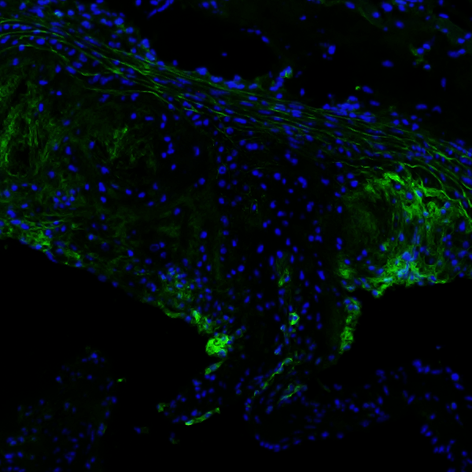

Supplement: Supplementary file 6 [file Data_Sheet_5.ZIP › macrophages/immunofluorescence/original figure/FO1.tif]

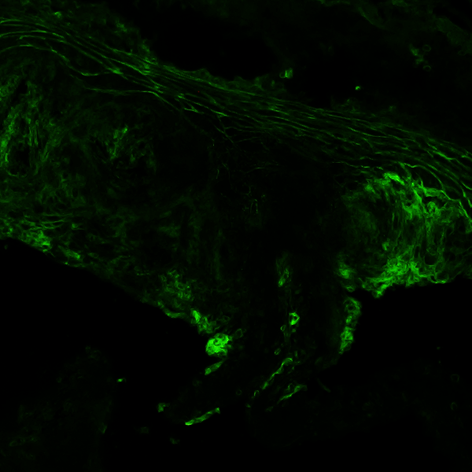

Supplement: Supplementary file 6 [file Data_Sheet_5.ZIP › macrophages/immunofluorescence/original figure/FO2.tif]

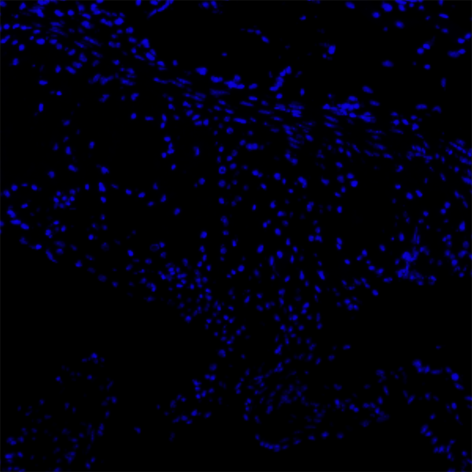

Supplement: Supplementary file 6 [file Data_Sheet_5.ZIP › macrophages/immunofluorescence/original figure/FO3.tif]

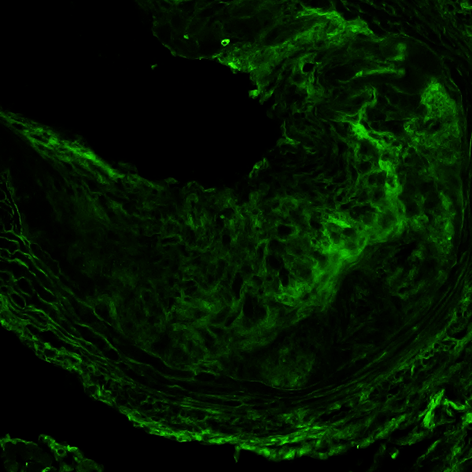

Supplement: Supplementary file 6 [file Data_Sheet_5.ZIP › macrophages/immunofluorescence/original figure/MOD 1.tif]

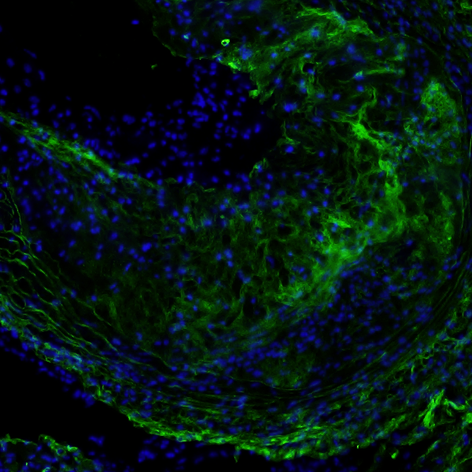

Supplement: Supplementary file 6 [file Data_Sheet_5.ZIP › macrophages/immunofluorescence/original figure/MOD2.tif]

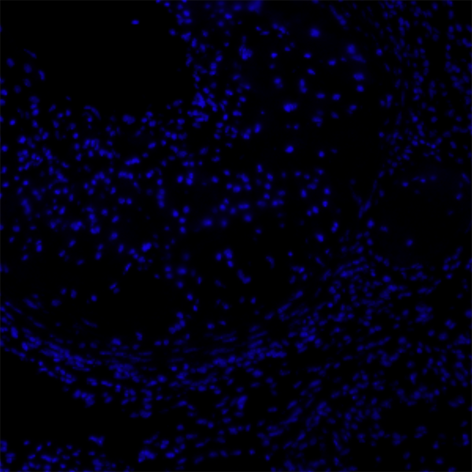

Supplement: Supplementary file 6 [file Data_Sheet_5.ZIP › macrophages/immunofluorescence/original figure/MOD3.tif]

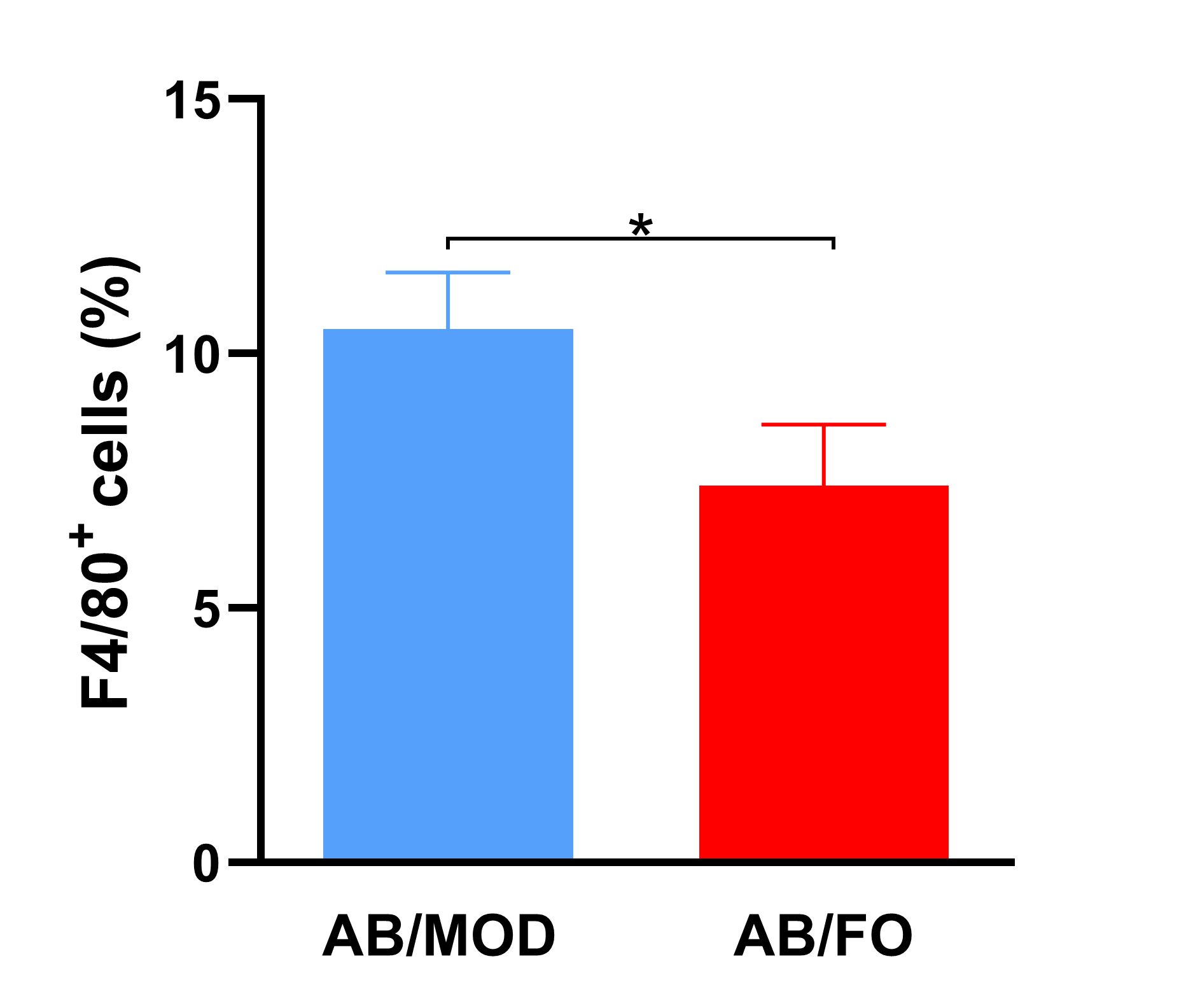

Supplement: Supplementary file 8 [file Data_Sheet_7.ZIP › macrophages/FLOW/AB-F480.tif]

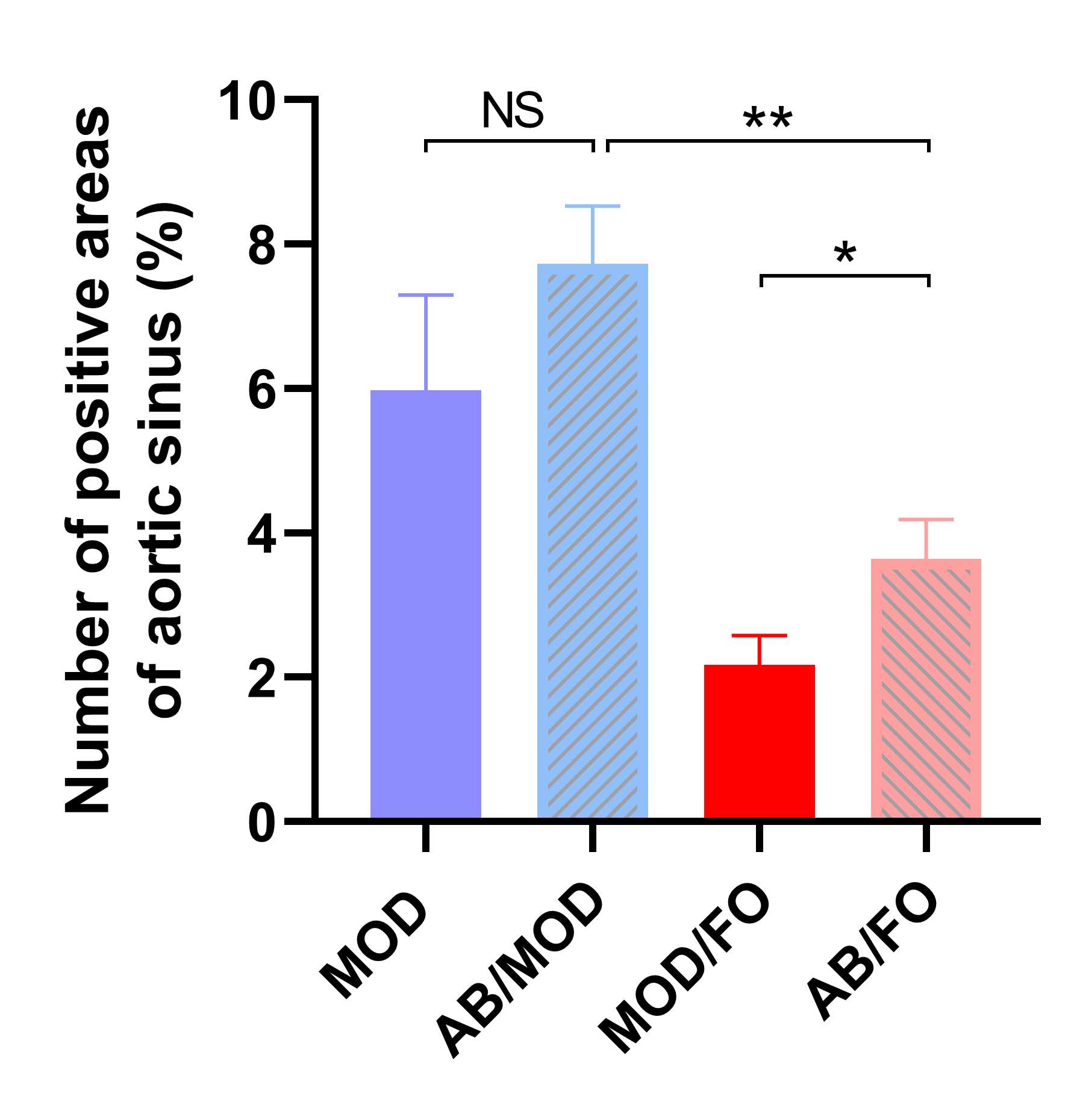

Supplement: Supplementary file 8 [file Data_Sheet_7.ZIP › macrophages/immunofluorescence/F480.tif]

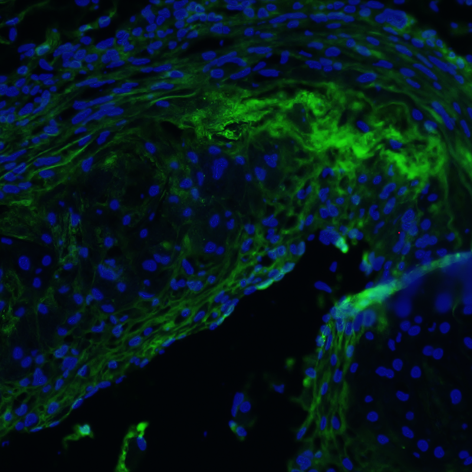

Supplement: Supplementary file 8 [file Data_Sheet_7.ZIP › macrophages/immunofluorescence/FO1.tif]

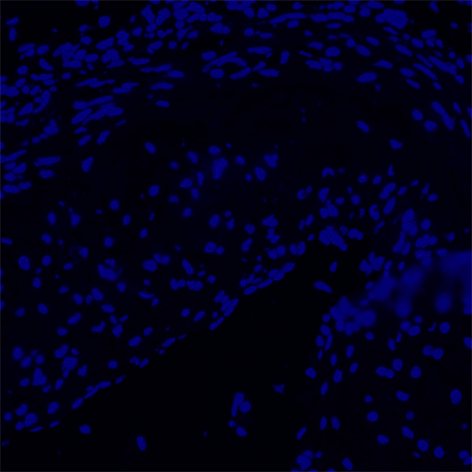

Supplement: Supplementary file 8 [file Data_Sheet_7.ZIP › macrophages/immunofluorescence/FO2.tif]

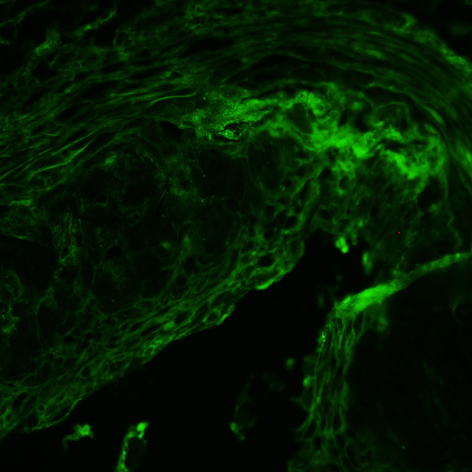

Supplement: Supplementary file 8 [file Data_Sheet_7.ZIP › macrophages/immunofluorescence/FO3.tif]

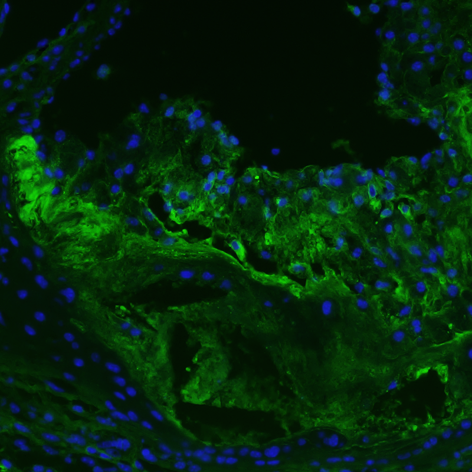

Supplement: Supplementary file 8 [file Data_Sheet_7.ZIP › macrophages/immunofluorescence/MOD1.tif]

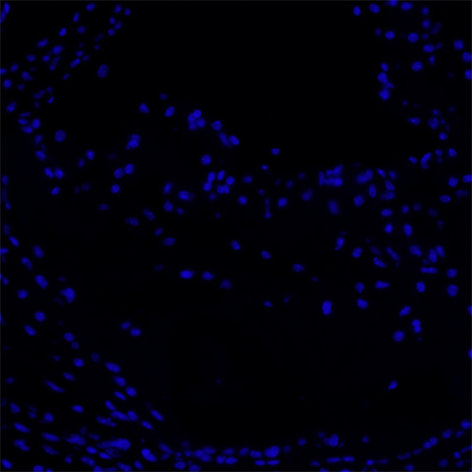

Supplement: Supplementary file 8 [file Data_Sheet_7.ZIP › macrophages/immunofluorescence/MOD2.tif]

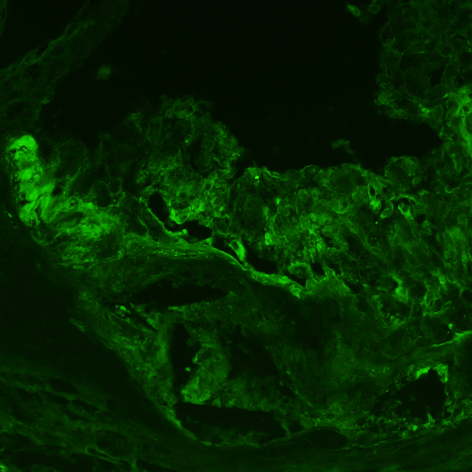

Supplement: Supplementary file 8 [file Data_Sheet_7.ZIP › macrophages/immunofluorescence/MOD3.tif]

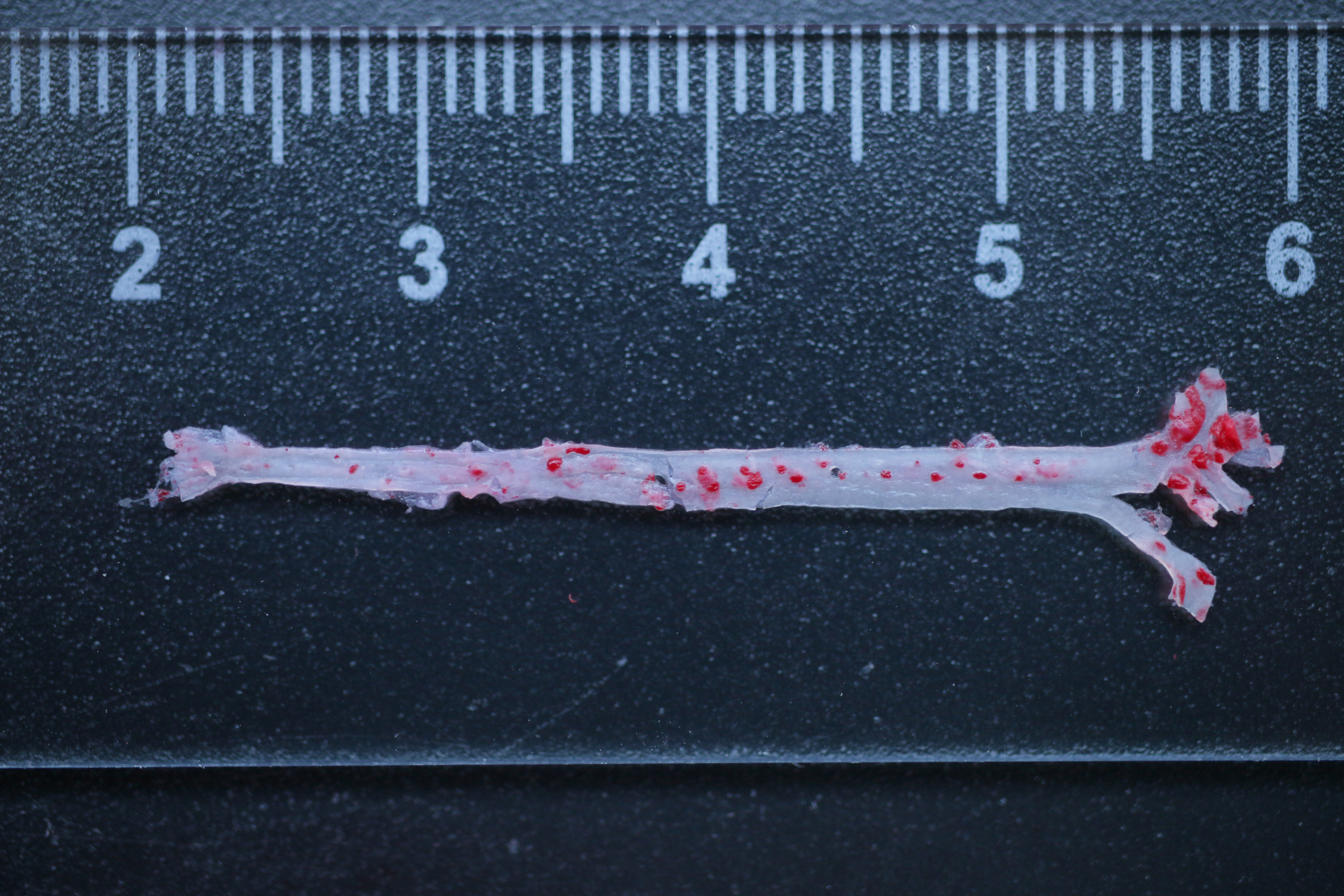

Supplement: Supplementary file 9 [file Data_Sheet_8.ZIP › pathological staining/antibiotic-original figure/AB+FO En face oil red O.JPG]

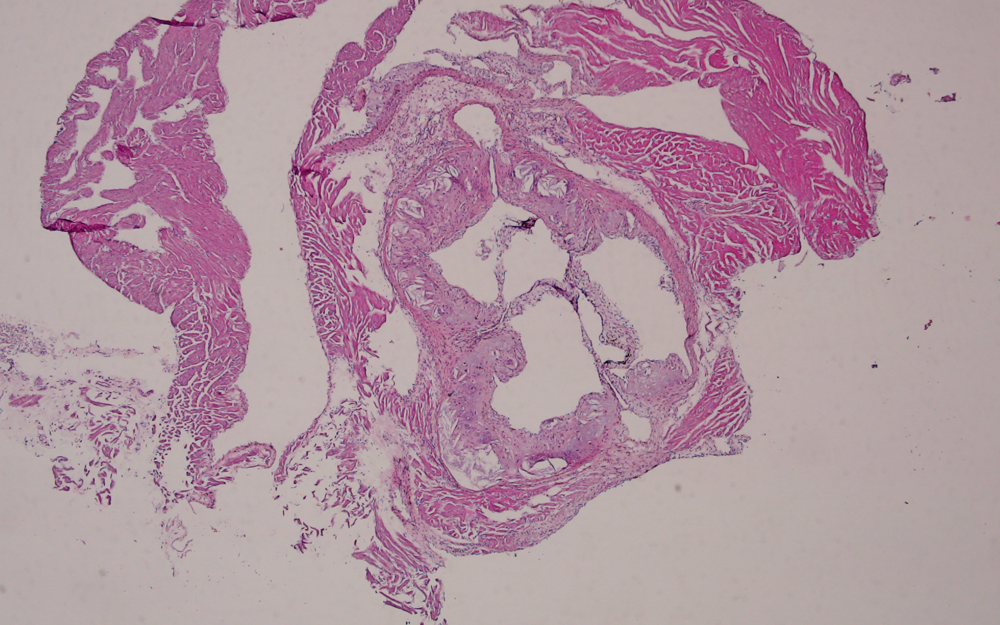

Supplement: Supplementary file 9 [file Data_Sheet_8.ZIP › pathological staining/antibiotic-original figure/AB+FO HE.tif]

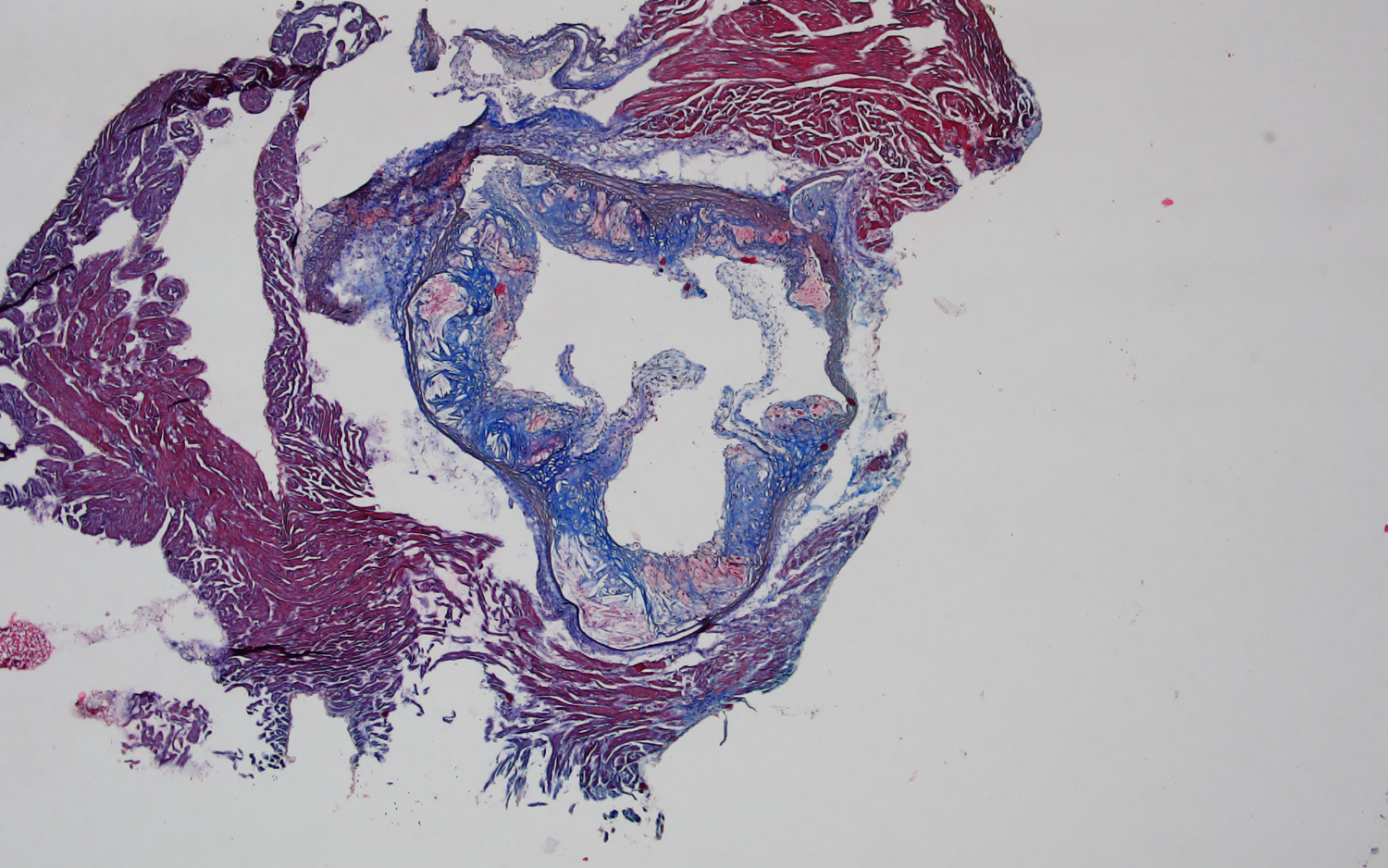

Supplement: Supplementary file 9 [file Data_Sheet_8.ZIP › pathological staining/antibiotic-original figure/AB+FO Masson.tif]

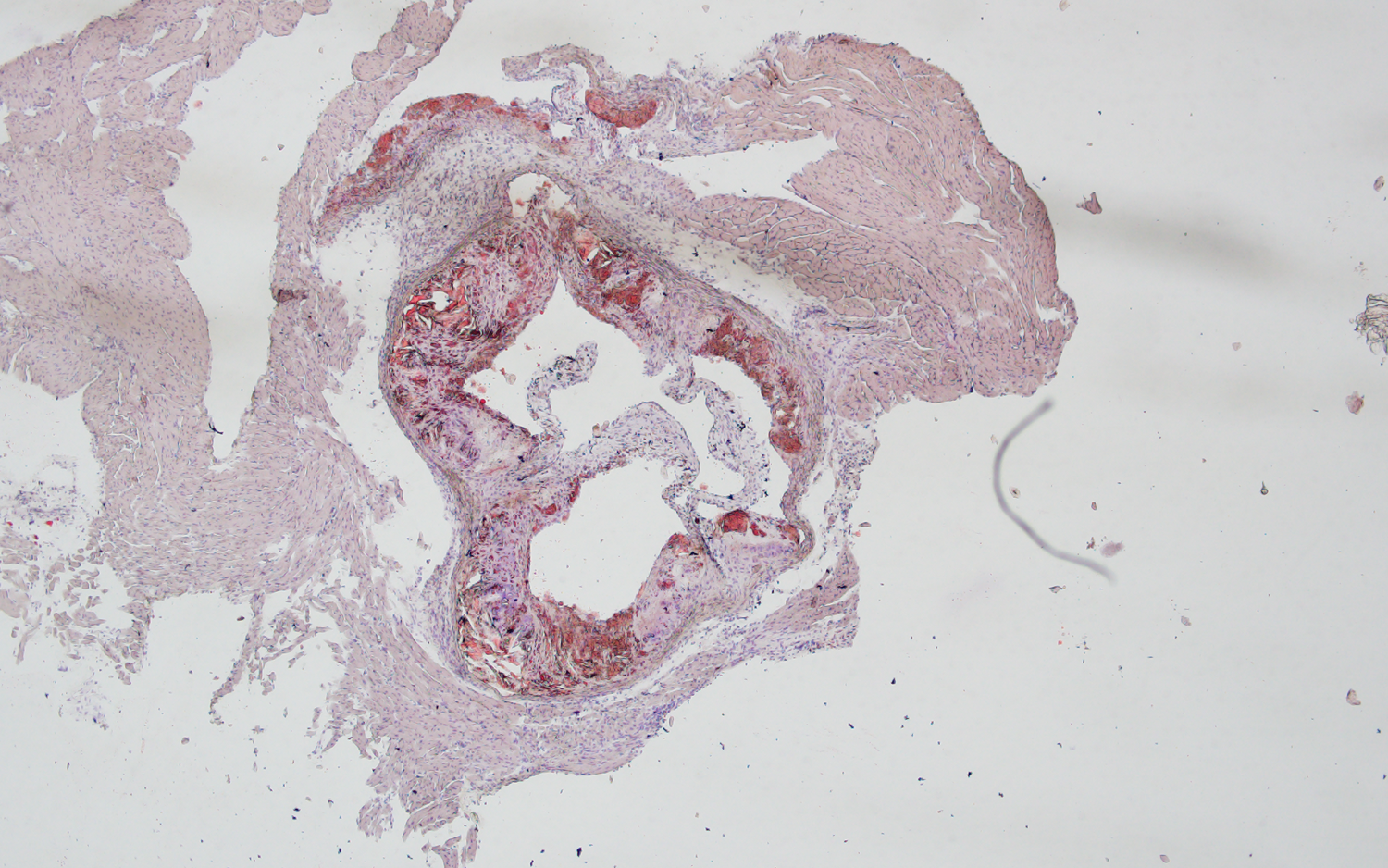

Supplement: Supplementary file 9 [file Data_Sheet_8.ZIP › pathological staining/antibiotic-original figure/AB+FO oil red O.tif]

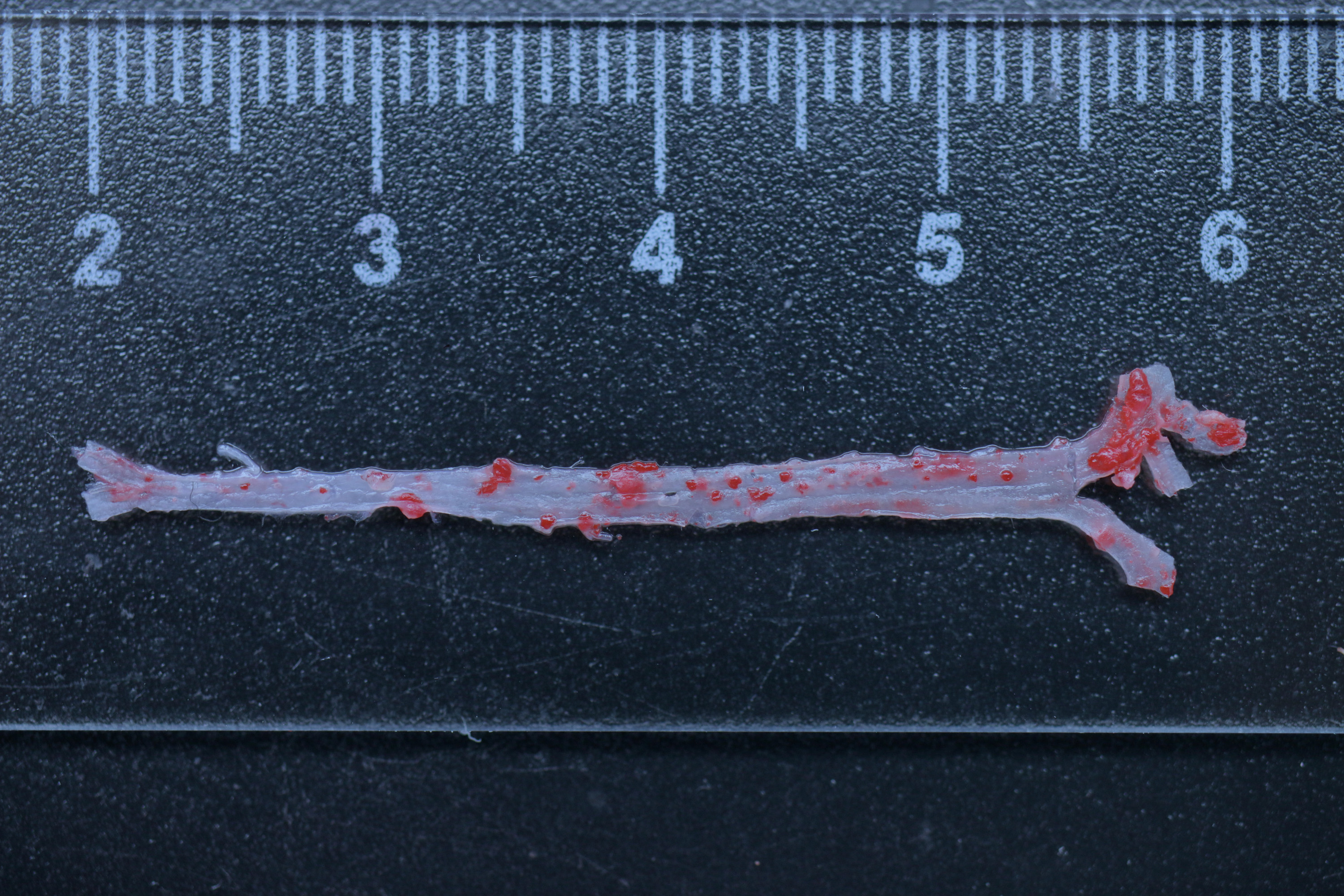

Supplement: Supplementary file 9 [file Data_Sheet_8.ZIP › pathological staining/antibiotic-original figure/AB+MOD En face oil red O.JPG]

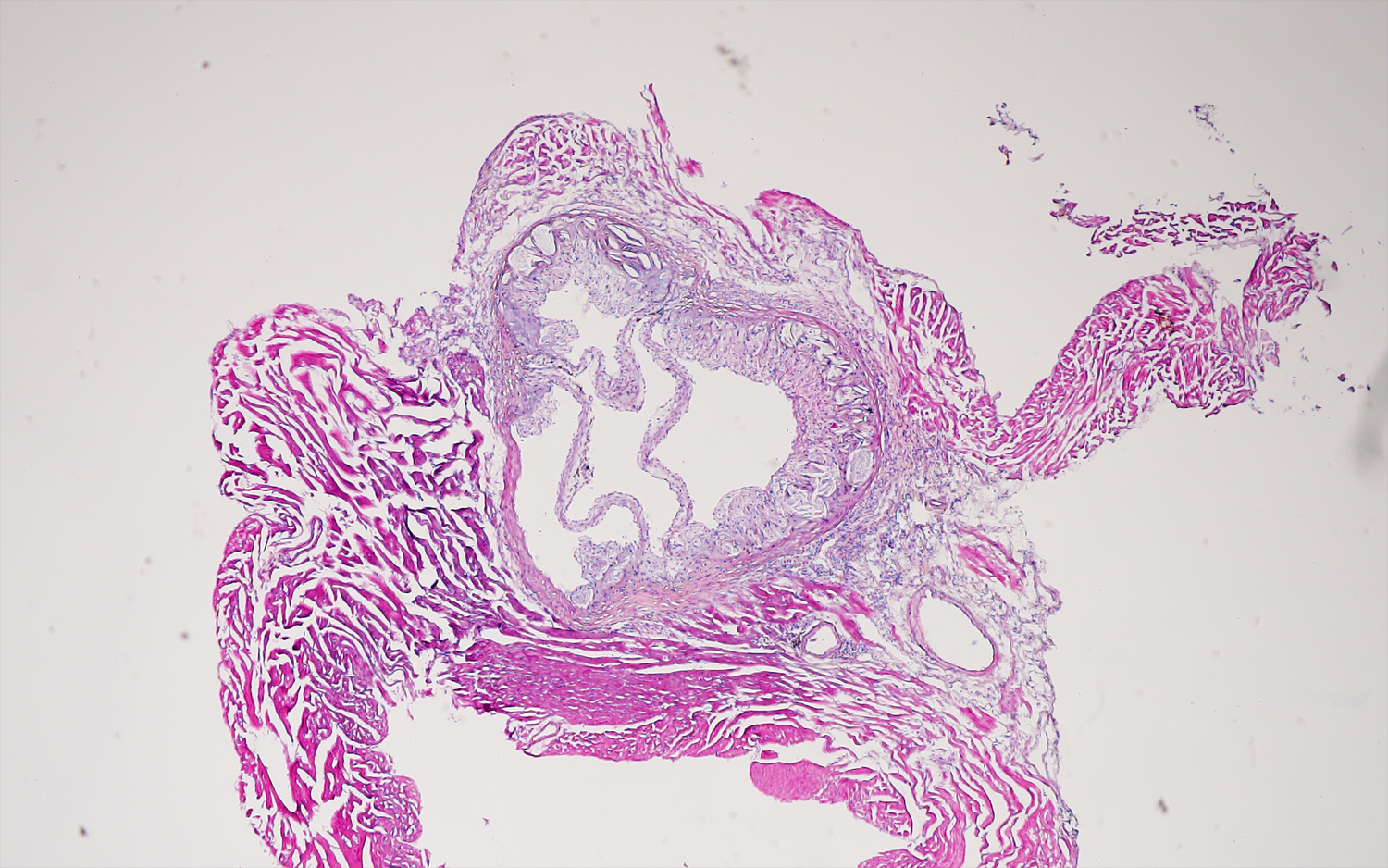

Supplement: Supplementary file 9 [file Data_Sheet_8.ZIP › pathological staining/antibiotic-original figure/AB+MOD-HE.tif]

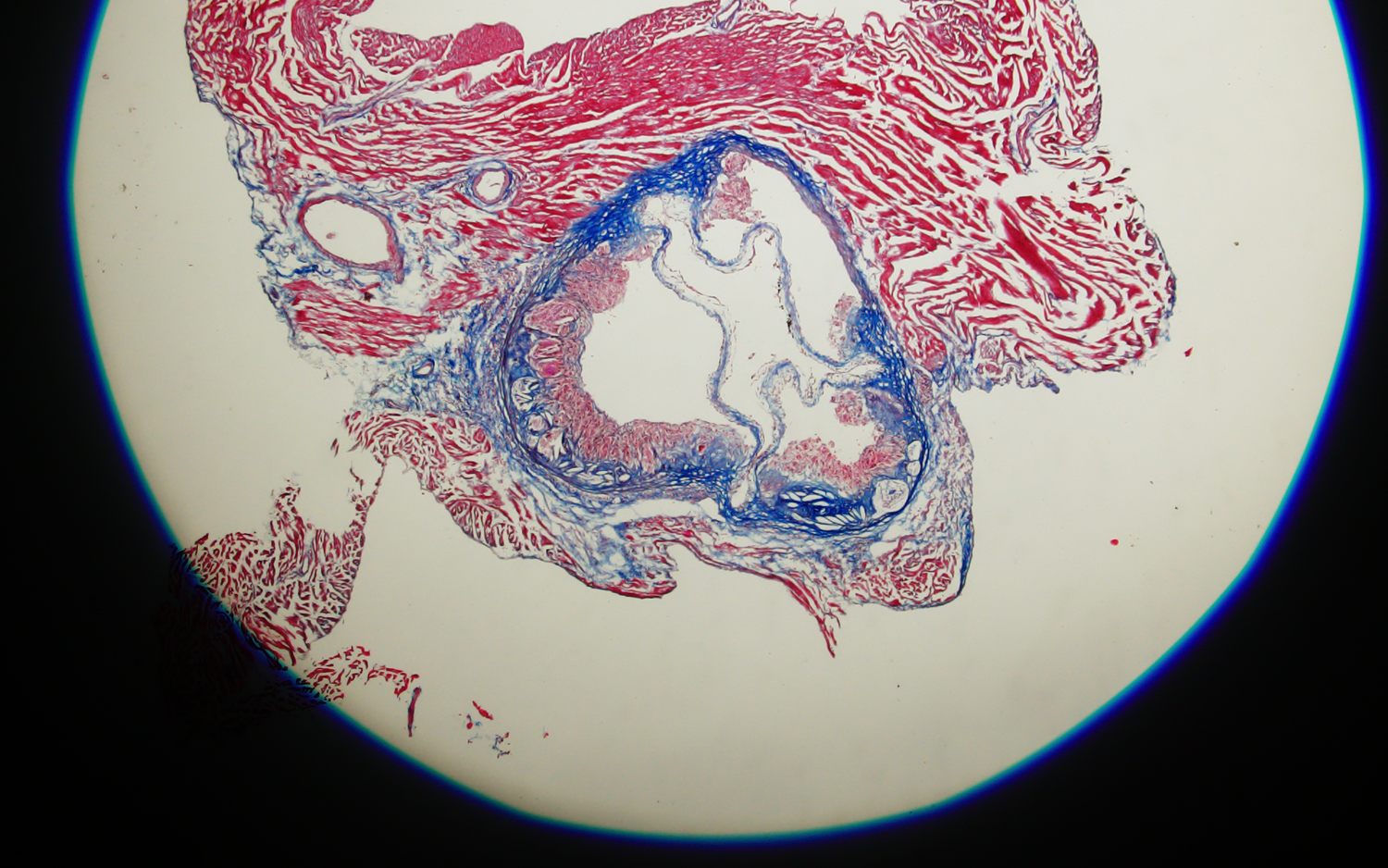

Supplement: Supplementary file 9 [file Data_Sheet_8.ZIP › pathological staining/antibiotic-original figure/AB+MOD-Masson.tif]

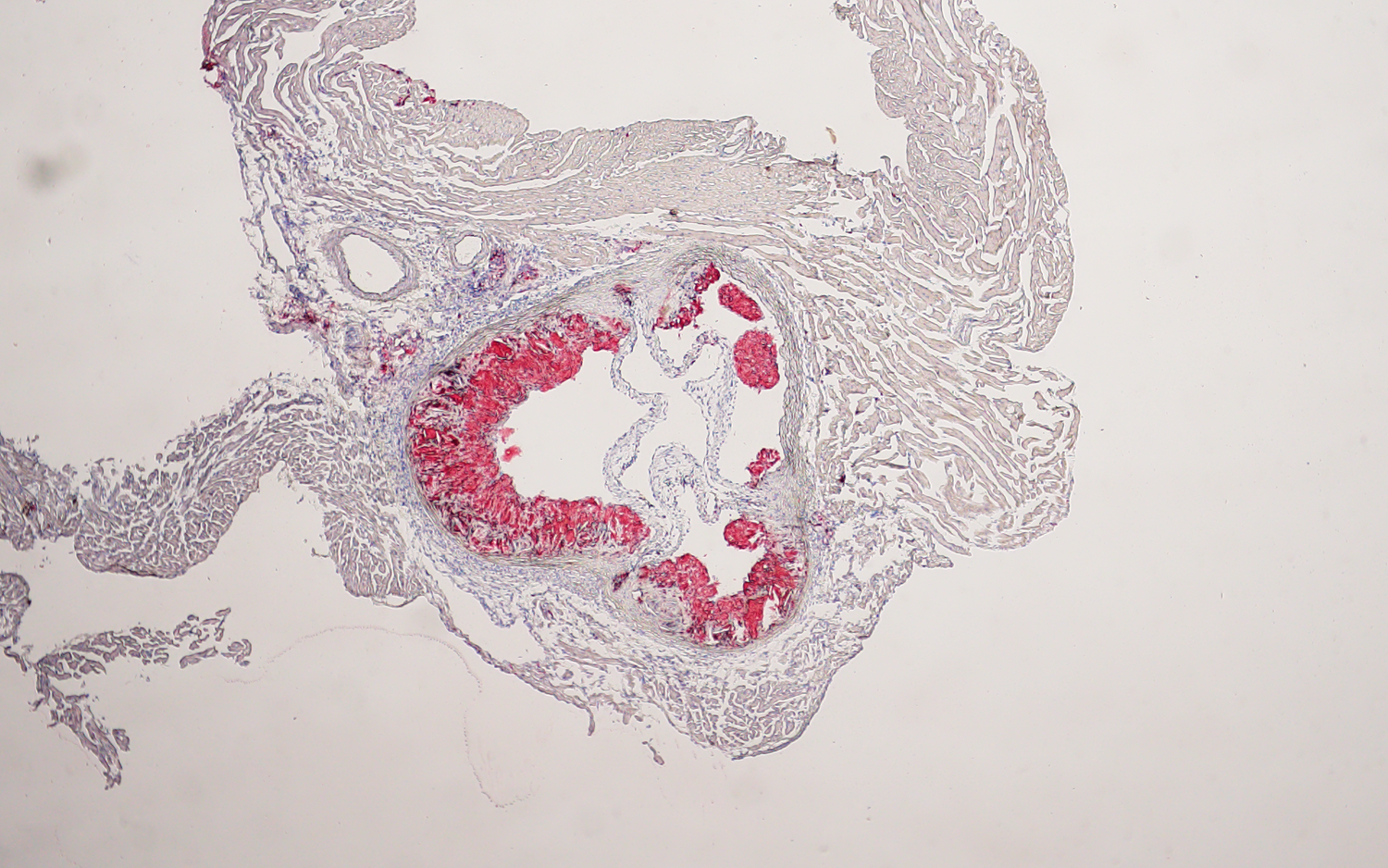

Supplement: Supplementary file 9 [file Data_Sheet_8.ZIP › pathological staining/antibiotic-original figure/AB+MOD-oil red O.tif]

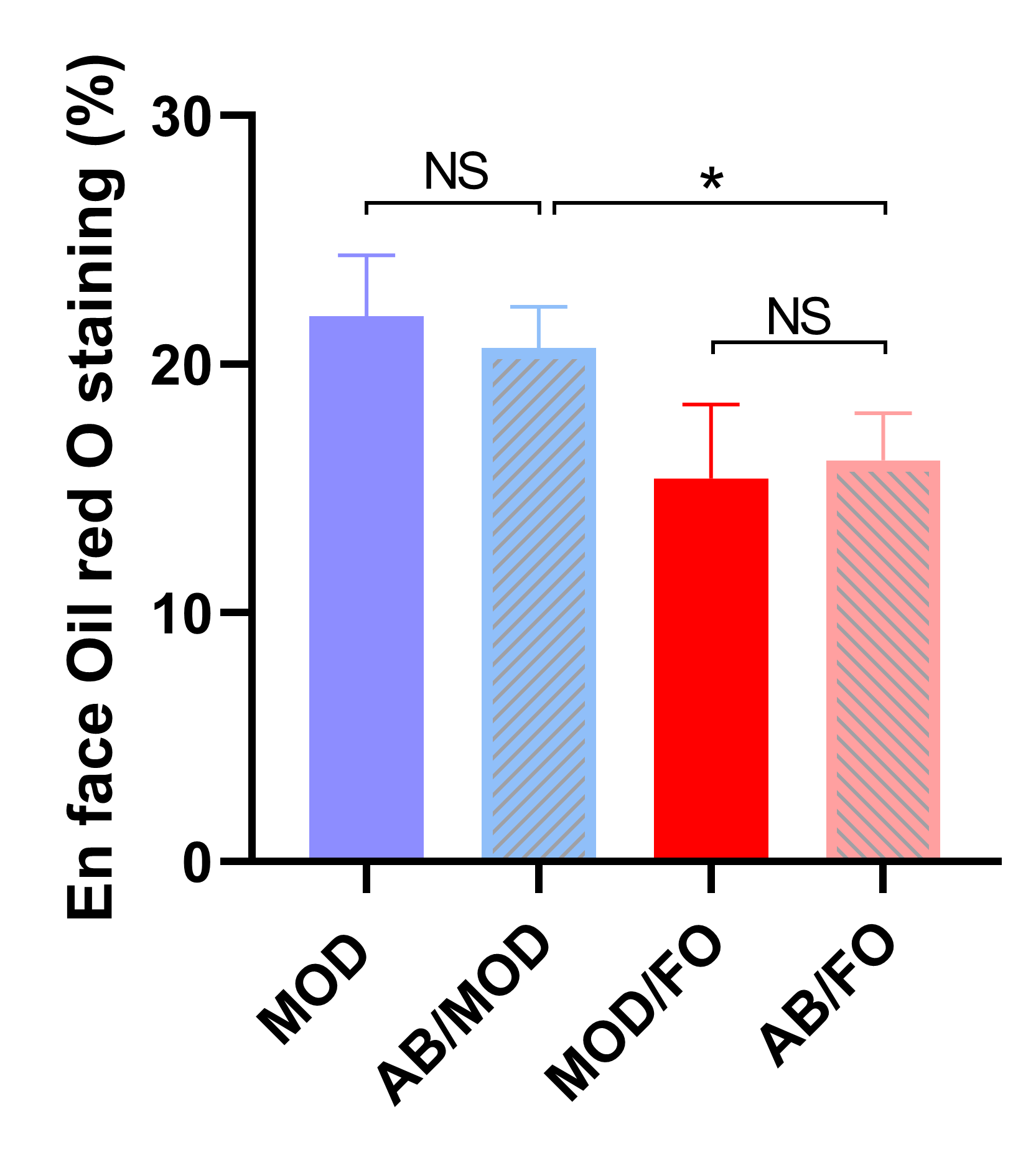

Supplement: Supplementary file 9 [file Data_Sheet_8.ZIP › pathological staining/en face oil red O/AB-En face oil red O.tif]

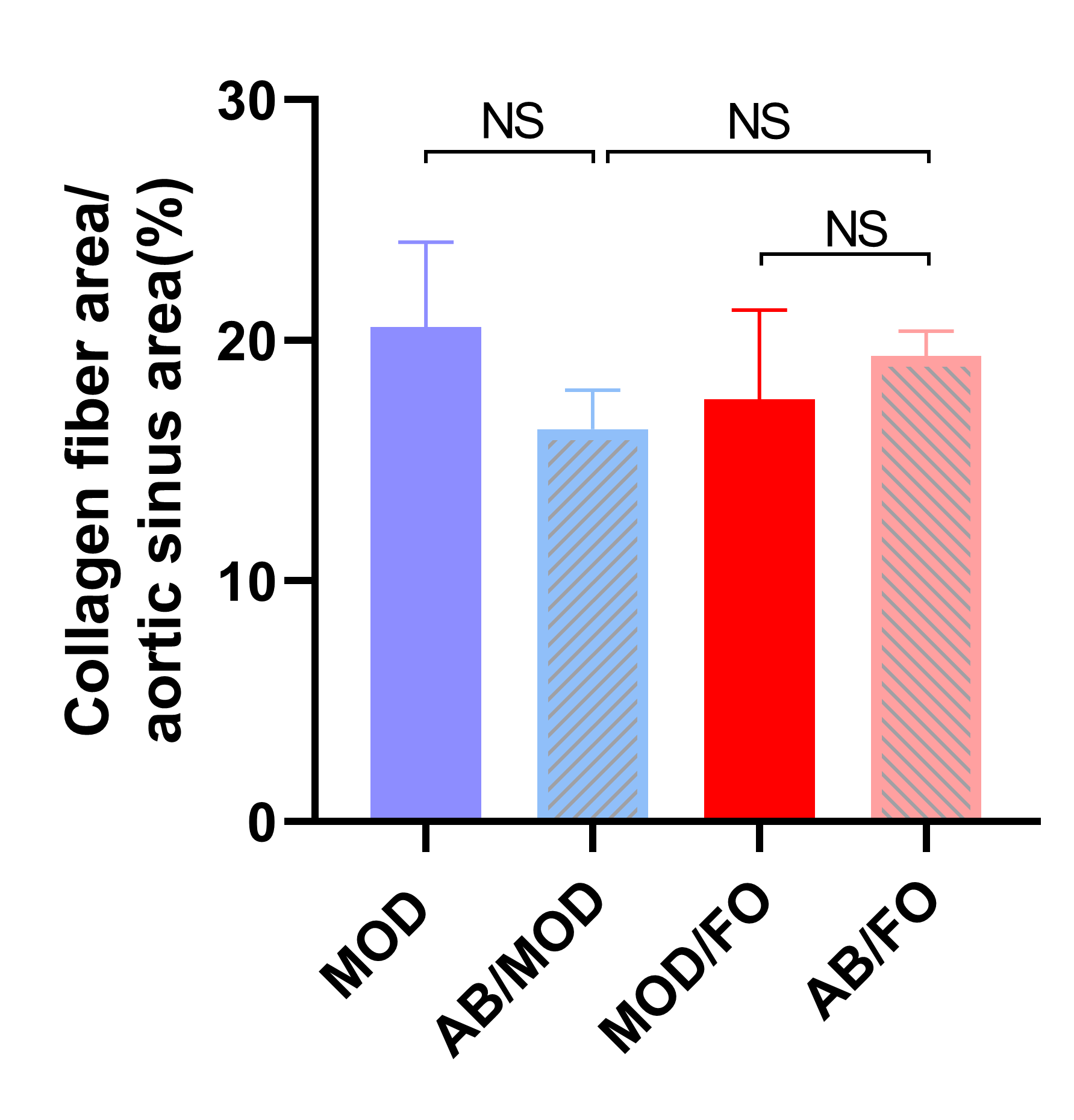

Supplement: Supplementary file 9 [file Data_Sheet_8.ZIP › pathological staining/Masson/AB-Masson.tif]

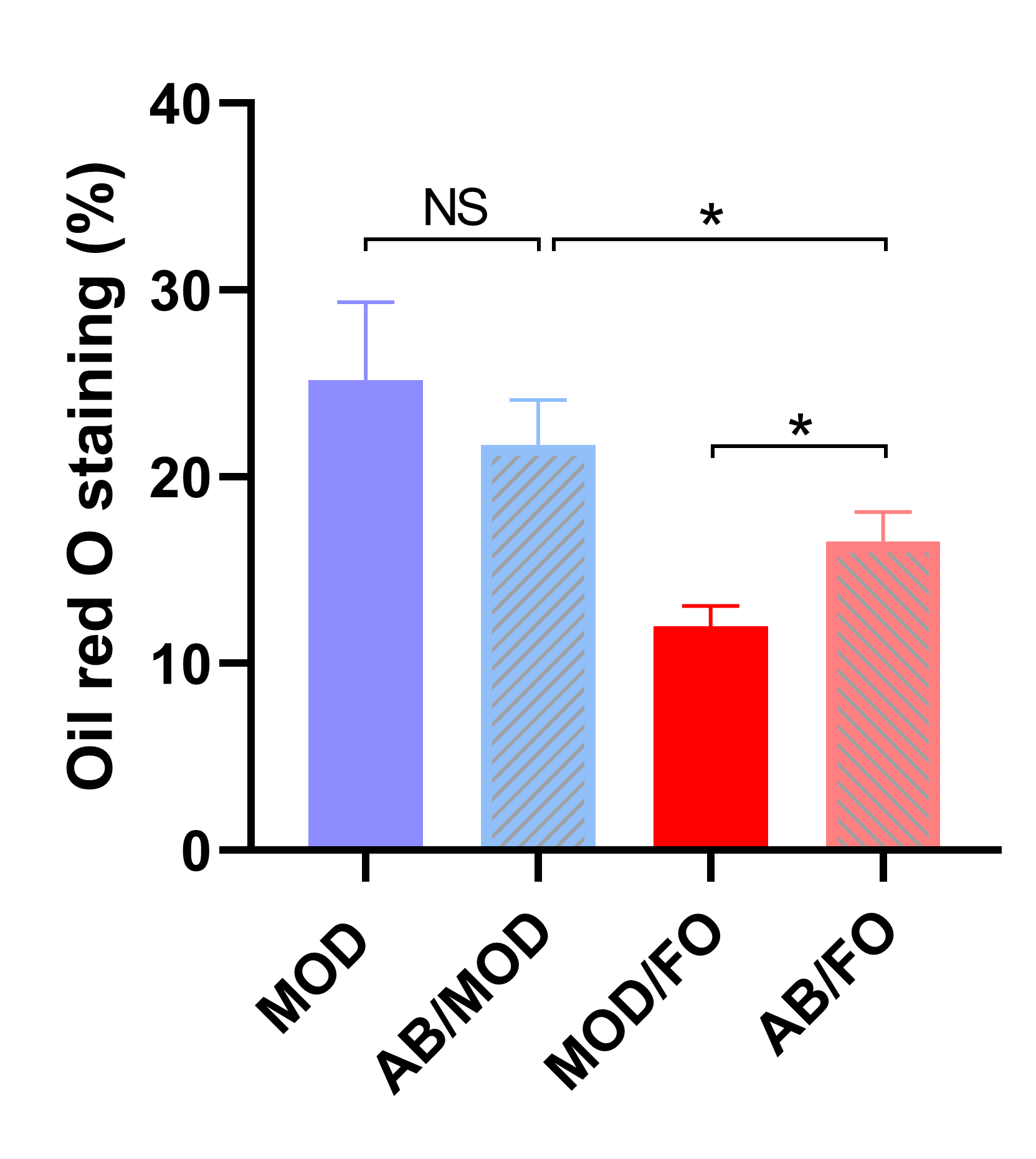

Supplement: Supplementary file 9 [file Data_Sheet_8.ZIP › pathological staining/oil red O/AB- oil red O.tif]
