# Supplementary material for: Dietary α-Linolenic Acid-Rich Flaxseed Oil Ameliorates High-Fat Diet-Induced Atherosclerosis via Gut Microbiota-Inflammation-Artery Axis in ApoE−/− Mice
Source: Front Cardiovasc Med. 2022 Feb 28;9:830781. doi: 10.3389/fcvm.2022.830781 (PMC8918482; doi:10.3389/fcvm.2022.830781)
Supplement: Supplementary file 7 [file Data_Sheet_6.ZIP › NCBI.rtf]

The raw reads of gut microbiota in all groups were submitted in NCBI SRA with an accession number PRJNA624814
